# An Emodin-Depot Microsphere-in-Hydrogel Reprograms the Immuno-myogenic Niche to Enable Volumetric Muscle Loss Repair Revealed by Single-Cell Profiling

**DOI:** 10.34133/research.1329

**Published:** 2026-08-12

**Authors:** Wanshun Liu, Ruizhe Wang, Fu Zhao, Zhengyuan Fang, Jun Ma, Zhixuan Mai, Yanwei He, Junzhe Sheng, Yunxuan Shi, Zhijie Zhao, Hongling Jia, Xiaojing Wang, Wei Luo, Renwen Wan, Shiyi Chen, Gang Chen, Qi Sun, Zhiwen Luo, Xinming Ye, Nirong Bao, Xiaochuan Gu

**Affiliations:** ^1^ Department of Sports Medicine, Nanjing Hospital of Chinese Medicine, Nanjing University of Chinese Medicine, Nanjing, China.; ^2^ Spine Center, Department of Orthopedics, Shanghai Changzheng Hospital, Naval Medical University, Shanghai 200003, China.; ^3^ School of Traditional Chinese Medicine, Jinan University, Guangzhou, China.; ^4^ Department of Gastrointestinal Surgery, Renji Hospital, School of Medicine, Shanghai Jiao Tong University, Shanghai, China.; ^5^ Department of Orthopaedics, Jiaxing Key Laboratory of Basic Research and Clinical Translation on Orthopedic Biomaterials, The Second Affiliated Hospital of Jiaxing University, Jiaxing 314000, China.; ^6^ Fudan University–Dr. Kong Joint Research Center for Sports Medicine and Health Footwear, Fudan University Institute of Sports Medicine (Jinqiao Laboratory), Zhangjiang Institute, Fudan University, Shanghai, China.; ^7^ Department of Sports Medicine, Huashan Hospital, Fudan University, Shanghai 200040, China.; ^8^ College of First Clinical Medicine, Shandong University of Traditional Chinese Medicine, Jinan 250014, China.; ^9^ Department of Plastic and Reconstructive Surgery, Shanghai Ninth People’s Hospital, Shanghai Jiao Tong University School of Medicine, Shanghai, China.; ^10^ Department of Rheumatology and Immunology, Tongren Hospital, School of Medicine, Shanghai Jiao Tong University, Shanghai, China.; ^11^ School of Sports Science and Engineering/Research Institute of Health Science and Engineering, East China University of Science and Technology, Shanghai 200237, China.; ^12^ Department of Orthopedics, Nanjing Jinling Hospital, Affiliated Hospital of Medical School, Nanjing University, Nanjing, China.; ^13^ Department of Orthopedics, Shanghai Changhai Hospital, Naval Medical University, Shanghai 200433, China.

## Abstract

Volumetric muscle loss (VML) causes irreversible loss of contractile tissue and creates a hostile regenerative niche marked by sustained inflammation, oxidative stress, fibrosis, and poor functional recovery. Here, we develop an injectable and photocurable microsphere-in-hydrogel platform that couples structural support with sustained small-molecule immunoregulation. To counteract this hostile microenvironment, we utilized emodin, a natural anthraquinone recognized for its potent anti-inflammatory and reactive-oxygen-species-scavenging properties. Emodin-loaded sodium alginate microspheres were generated via ionic crosslinking and embedded within a gelatin methacryloyl matrix to form E-AMs@GM. The composite hydrogel exhibited defect-conforming moldability, porous microarchitecture, tunable swelling/degradation, and broad interfacial adhesion. In vitro, E-AMs@GM showed excellent cytocompatibility and attenuated intracellular reactive oxygen species in human bone-marrow-derived mesenchymal stem cells under oxidative challenge. In macrophages, E-AMs@GM reduced pro-inflammatory activation while enhancing pro-regenerative programs, accompanied by decreased inflammatory cytokines and increased interleukin-10. E-AMs@GM also promoted C2C12 myogenic differentiation and myotube maturation. In a murine VML model, E-AMs@GM alleviated inflammation and fibrotic remodeling, increased myogenic progenitor activity and myofiber regeneration, and improved locomotor performance by CatWalk analysis. Mechanistically, scRNA sequencing revealed that E-AMs@GM enriches a reparative *Mmp12*^*+*^ macrophage subset and rewires macrophage–muscle satellite cell interactions through SPP1–CD44 and SPP1–integrin signaling, consistent with accelerated transition from inflammatory clearance to tissue reconstruction. Together, this depot-enabled hydrogel provides an instructive biomaterial strategy for functional VML repair.

## Introduction

Volumetric muscle loss (VML) represents a devastating form of skeletal muscle injury characterized by the irrecoverable loss of muscle mass and architecture beyond the endogenous regenerative capacity of the tissue [1,2]. Unlike minor strains that heal via satellite-cell-mediated regeneration, VML involves the extensive destruction of the extracellular matrix (ECM) and the resident progenitor microenvironment [3–5]. Current clinical treatments, such as autologous muscle transfer and functional flaps, can provide tissue coverage but are frequently limited by donor-site morbidity, incomplete innervation, and pervasive fibrotic scarring [6–9]. Consequently, there is an urgent need for regenerative strategies that not only fill the defect volume but also actively reprogram the local microenvironment to support durable functional recovery [10].

A central barrier to effective VML repair is the dysregulated inflammatory cascade that follows large-volume tissue loss [1,4]. In VML, persistent inflammation and excessive oxidative stress create a hostile niche that disrupts the regenerative orchestrations between macrophages and myogenic progenitors [2,9,11]. Specifically, the accumulation of pro-inflammatory (M1) macrophages and reactive oxygen species (ROS) impairs myogenic differentiation while promoting the activation of fibro-adipogenic progenitors [1,2]. This maladaptive signaling shifts the repair trajectory toward the formation of stiff, collagen-rich scar tissue rather than functional myofibers [12,13]. Therefore, successful VML repair requires a strategy that provides structural support while simultaneously suppressing oxidative inflammation and restoring a pro-regenerative (M2) immune niche [14–18].

Biomaterial scaffolds, particularly gelatin methacryloyl (GelMA) hydrogels, offer a compelling platform for VML repair due to their injectability, photocurable capability, and intrinsic cell-adhesive properties [19–21]. However, while GelMA provides a permissive physical template, it lacks the inherent bioactivity to counteract the severe inflammatory and oxidative environment of VML [22–25]. Emodin, a naturally occurring anthraquinone, possesses potent anti-inflammatory and antioxidative activities that are mechanistically well suited for niche reprogramming [26–28]. Among anthraquinone analogs, aloe-emodin is unsuitable for implantable applications due to its pronounced photosensitizing properties [29], and rhein carries cytotoxic liability at tissue-relevant concentrations [30,31]. Emodin, by contrast, exerts light-independent immunomodulatory effects through bidirectional regulation of macrophage polarization via nuclear factor kappa B/interferon regulatory factor 5/signal transducer and activator of transcription 1 and interferon regulatory factor 4/signal transducer and activator of transcription 6 signaling [32] and scavenges ROS through both nuclear factor erythroid 2-related factor 2-mediated enzymatic pathways and direct phenolic hydroxyl-mediated radical quenching [33–37]. Nevertheless, the therapeutic application of emodin is constrained by its poor aqueous solubility and rapid clearance, necessitating a controlled delivery system to ensure sustained local efficacy [38–41].

To address these challenges, we engineered an injectable, photocurable microsphere-in-hydrogel system in which emodin-loaded alginate microspheres serve as sustained-release depots embedded within a GelMA scaffold. This hierarchical design combines the defect-conforming capacity of GelMA with the prolonged immunomodulatory effects of emodin, thereby minimizing burst release and improving drug retention. In this study, we investigated the ability of this composite system to coordinately modulate the immune–myogenic microenvironment, aiming to suppress maladaptive inflammation, promote pro-regenerative macrophage polarization, and facilitate functional muscle regeneration with reduced fibrosis.

## Results

### Rational design and physicochemical characterization of the emodin-depot microsphere-in-hydrogel system

**Fig. 1.**
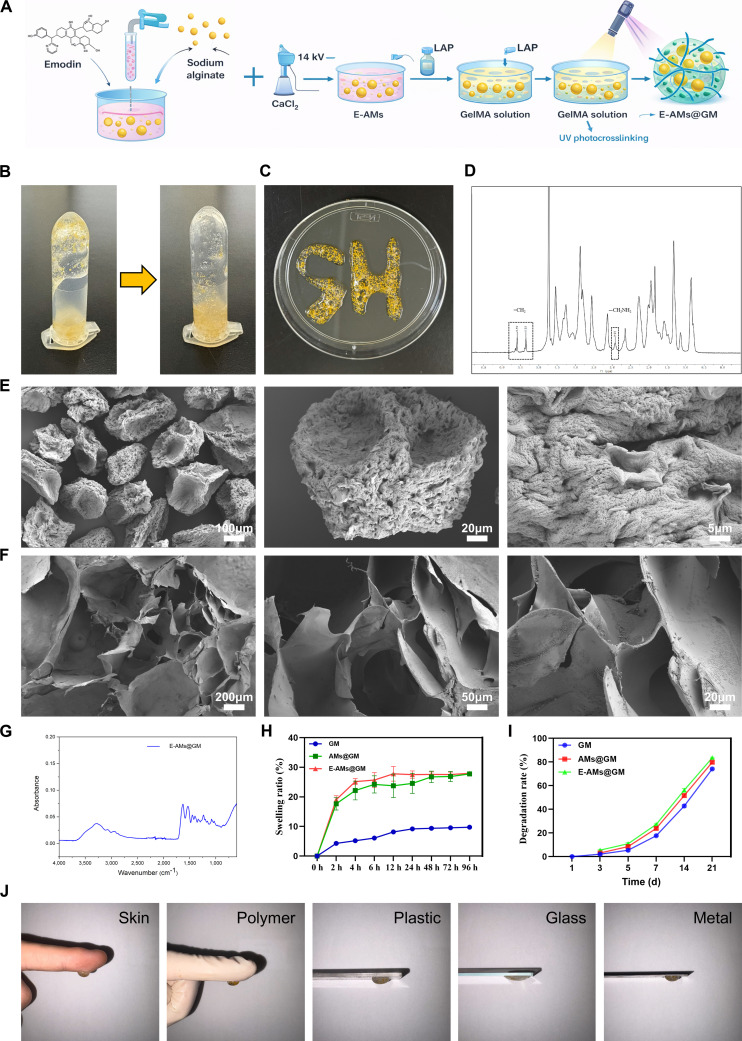
Design, fabrication, and physicochemical characterization of the emodin-depot microsphere-in-hydrogel system (emodin-loaded sodium alginate microspheres embedded within a gelatin methacryloyl matrix, E-AMs@GM). (A) Schematic illustration of the fabrication process: emodin is encapsulated into ionically crosslinked sodium alginate microspheres (E-AMs) via electrostatic spraying into CaCl_2_, followed by dispersion of E-AMs in gelatin methacryloyl (GelMA) precursor containing LAP and in situ photocrosslinking to form the composite hydrogel (E-AMs@GM). (B) Photographs demonstrating the macroscopic formation and microsphere distribution within the GelMA matrix before and after gelation, indicating stable entrapment of microspheres within the hydrogel network. (C) Shape conformability/moldability of the hydrogel, exemplified by patterning into predefined shapes. (D) Chemical functionalization analysis confirming GelMA methacrylation (representative spectrum shown). (E) Representative scanning electron microscopy (SEM) images of drug-loaded alginate microspheres at increasing magnifications. (F) Representative SEM images of the freeze-dried hydrogel showing the porous microarchitecture at different magnifications. (G) Absorbance spectrum (as indicated) of the hydrogel formulations, supporting successful incorporation of the drug depot within the matrix. (H) Swelling behavior of GM, AMs@GM, and E-AMs@GM over time. (I) In vitro degradation profiles of the hydrogels over the indicated period. (J) Qualitative adhesion assessment of the hydrogel to diverse substrates (skin, polymer, plastic, glass, and metal), demonstrating broad interfacial adhesiveness. Data are presented as mean ± SD (*n* = 3). Scale bars are indicated in the corresponding panels.

To tackle the pathological hallmarks of VML, we constructed a hierarchical microsphere-in-hydrogel platform (emodin-loaded sodium alginate microspheres embedded within a gelatin methacryloyl matrix, E-AMs@GM) that integrates defect-conforming structural support with sustained local immunomodulation. As illustrated in Fig. 1A, emodin-loaded sodium alginate microspheres (E-AMs) were produced by electrostatic spraying into a CaCl_2_ bath to achieve ionic crosslinking, followed by washing and lyophilization. The microspheres were then uniformly dispersed in a GelMA/lithium phenyl-2,4,6-trimethylbenzoylphosphinate (LAP) precursor and rapidly photocured in situ to form the composite hydrogel.

Macroscopic observations showed a homogeneous microsphere distribution within the GelMA network (Fig. 1B), and the material exhibited excellent shape fidelity and moldability (Fig. 1C), supporting its adaptability to irregular defect geometries. Successful methacrylation of the gelatin backbone was confirmed by proton nuclear magnetic resonance (Fig. 1D). Scanning electron microscopy (SEM) imaging demonstrated that E-AMs retained a spherical morphology after incorporation (Fig. 1E), while the freeze-dried hydrogel displayed a porous and interconnected microarchitecture (Fig. 1F) conducive to mass transport and cellular infiltration. The presence of emodin in the composite was further supported by UV–Vis absorbance spectra (Fig. 1G). Functionally, E-AMs@GM showed controlled swelling behavior and a time-dependent degradation profile consistent with progressive tissue remodeling requirements (Fig. 1H and I). In addition, the hydrogel exhibited robust interfacial adhesion to diverse substrates (tissue and common engineering materials), suggesting improved retention under the mechanically dynamic muscle environment (Fig. 1J).

### In vitro cytocompatibility and ROS-scavenging capacity of E-AMs@GM

**Fig. 2.**
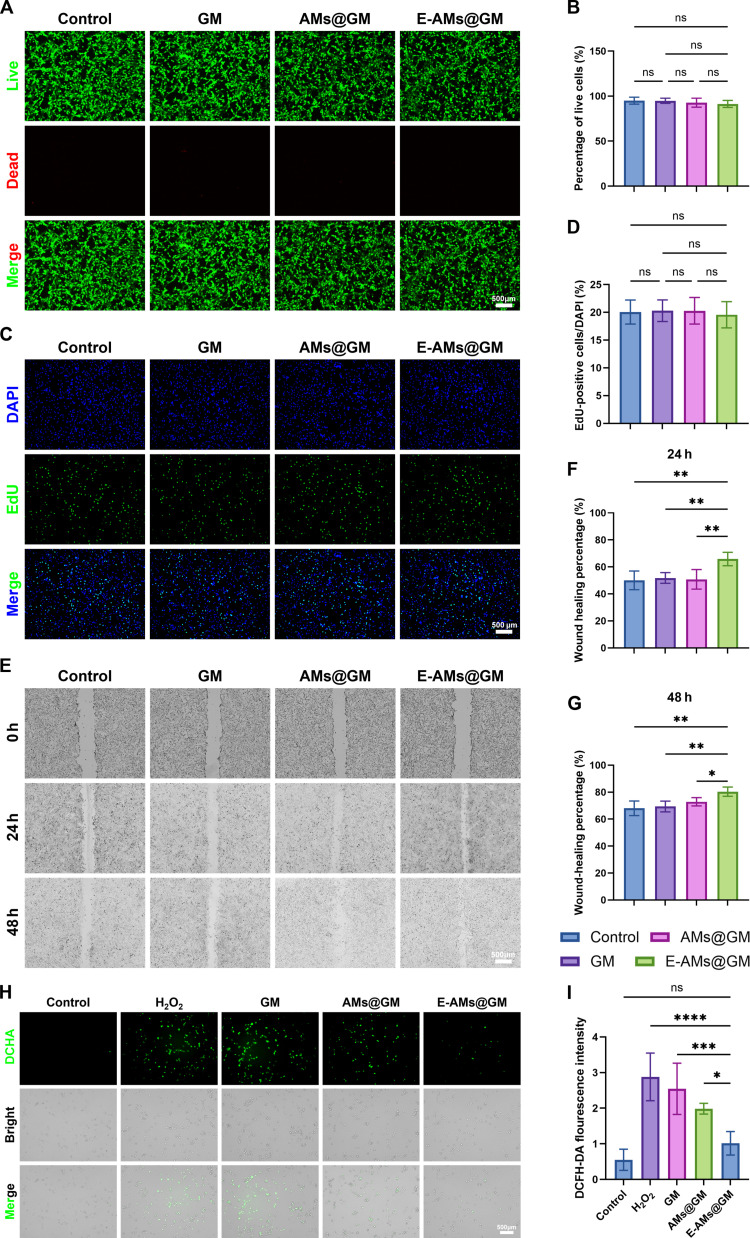
In vitro biocompatibility and pro-regenerative effects of E-AMs@GM. Human bone-marrow-derived mesenchymal stem cells (hBMSCs) were used to evaluate the biosafety and cellular responses to the hydrogels, including GM, AMs@GM, and E-AMs@GM. (A and B) Live/Dead staining of hBMSCs cultured with/within the indicated hydrogels, showing representative fluorescence images (A) and corresponding quantitative analysis of cell viability (B). (C and D) 5-Ethynyl-2′-deoxyuridine (EdU) incorporation assay assessing hBMSC proliferation, with representative images (C) and quantification of EdU-positive cells (D). DAPI, 4′,6-diamidino-2-phenylindole. (E to G) Scratch-wound-healing assay evaluating hBMSC migration, including representative images at the indicated time points (E), measurement of wound closure over time (F), and summary quantification of migration rate/closure percentage (G). (H and I) Oxidative-stress assessment of hBMSCs under the indicated conditions (e.g., H_2_O_2_ challenge), presenting representative 2′,7′-dichlorodihydrofluorescein diacetate (DCFH-DA) fluorescence images (H) and quantitative analysis of intracellular ROS/oxidative-stress level (I). Data are shown as mean ± SD (*n* = 5). ns, not significant; *P < 0.05, **P < 0.01, ***P < 0.001. Scale bars, 500 μm.

We next assessed biosafety and cellular compatibility using human bone-marrow-derived mesenchymal stem cells (hBMSCs). Live/Dead staining indicated high cell viability across GM, AMs@GM, and E-AMs@GM groups (Fig. 2A and B), demonstrating negligible cytotoxicity of the platform (viability >90%). 5-Ethynyl-2′-deoxyuridine incorporation assays further confirmed that the hydrogels supported normal proliferative activity (Fig. 2C and D).

Given that effective repair requires active cell recruitment and wound remodeling, we performed a scratch-wound-healing assay and found that hBMSCs exposed to E-AMs@GM displayed accelerated wound closure and enhanced migratory behavior relative to controls (Fig. 2E to G). Moreover, under H_2_O_2_-induced oxidative stress, E-AMs@GM markedly reduced intracellular ROS signals compared with GM and oxidative-stress-only conditions (Fig. 2H and I), supporting the concept that sustained emodin release protects reparative cells from oxidative injury and helps maintain a pro-regenerative microenvironment.

### E-AMs@GM reprograms macrophage polarization toward a pro-regenerative phenotype

**Fig. 3.**
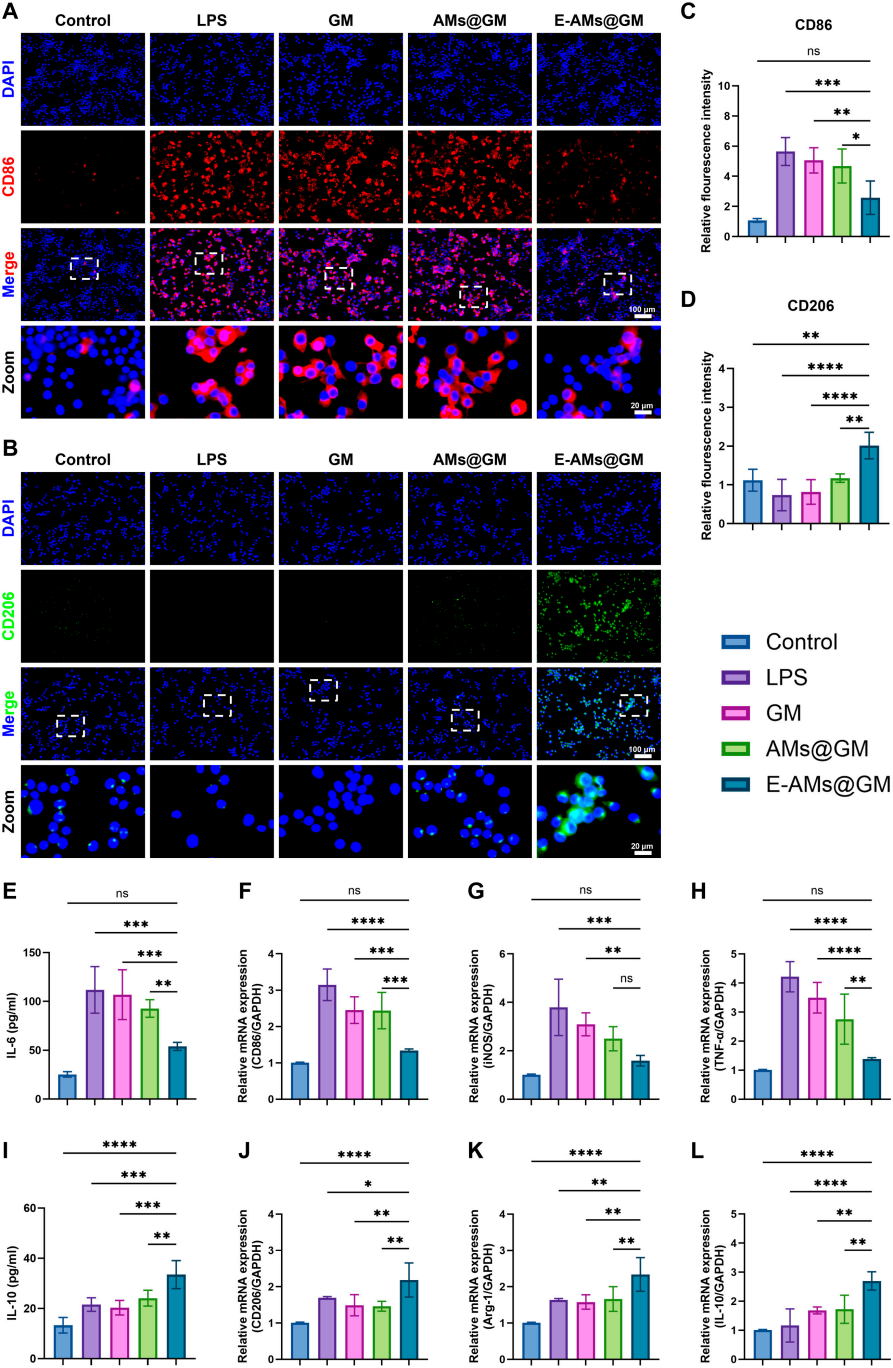
Immunomodulatory effects of E-AMs@GM on RAW 264.7 macrophage polarization and inflammatory responses. RAW 264.7 macrophages were cultured with GM, AMs@GM, or E-AMs@GM to assess immunoregulatory capacity. (A to D) Immunofluorescence staining of macrophage polarization markers, including CD86 (M1-associated; red) and CD206 (M2-associated; green), with nuclei counterstained by DAPI (blue). Representative low-magnification fields and corresponding high-magnification views are shown, together with quantitative analysis of CD86/CD206 fluorescence (as indicated). (E and I) Enzyme-linked immunosorbent assay quantification of cytokines in culture supernatants (pro-inflammatory and anti-inflammatory factors as labeled in the panels). (F to H and J to L) Reverse transcription–quantitative polymerase chain reaction analysis of polarization-related and inflammation-associated gene expression in RAW 264.7 cells following exposure to the different hydrogels; each panel corresponds to the indicated target gene. GAPDH, glyceraldehyde-3-phosphate dehydrogenase. Data are presented as mean ± SD (*n* = 5). ns, not significant; *P < 0.05, **P < 0.01, ***P < 0.001. Scale bars are indicated in the images.

Because macrophage polarization is a key determinant of regeneration versus fibrosis in VML, we evaluated immunoregulatory performance using RAW 264.7 macrophages. Immunofluorescence staining demonstrated that E-AMs@GM suppressed the M1-associated marker CD86 while enhancing the M2-associated marker CD206, indicating a shift toward a reparative macrophage phenotype (Fig. 3A to D).

These observations were supported by cytokine profiling: Enzyme-linked immunosorbent assay showed reduced secretion of pro-inflammatory cytokines (e.g., interleukin-6 and tumor necrosis factor-alpha [TNF-α]) and increased production of anti-inflammatory interleukin-10 in the E-AMs@GM group (Fig. 3E and I). Consistently, reverse transcription–quantitative polymerase chain reaction confirmed down-regulation of M1-associated transcripts (e.g., CD86, inducible nitric oxide synthase [iNOS], and TNF-α) and up-regulation of M2-associated genes (e.g., CD206, Arg-1, and interleukin-10) (Fig. 3F to H and J to L). Collectively, these data indicate that E-AMs@GM acts as an immunomodulatory scaffold, actively biasing macrophage programs away from sustained inflammation and toward regeneration-supportive functions.

### E-AMs@GM enhances myogenic differentiation and myotube maturation in vitro

**Fig. 4.**
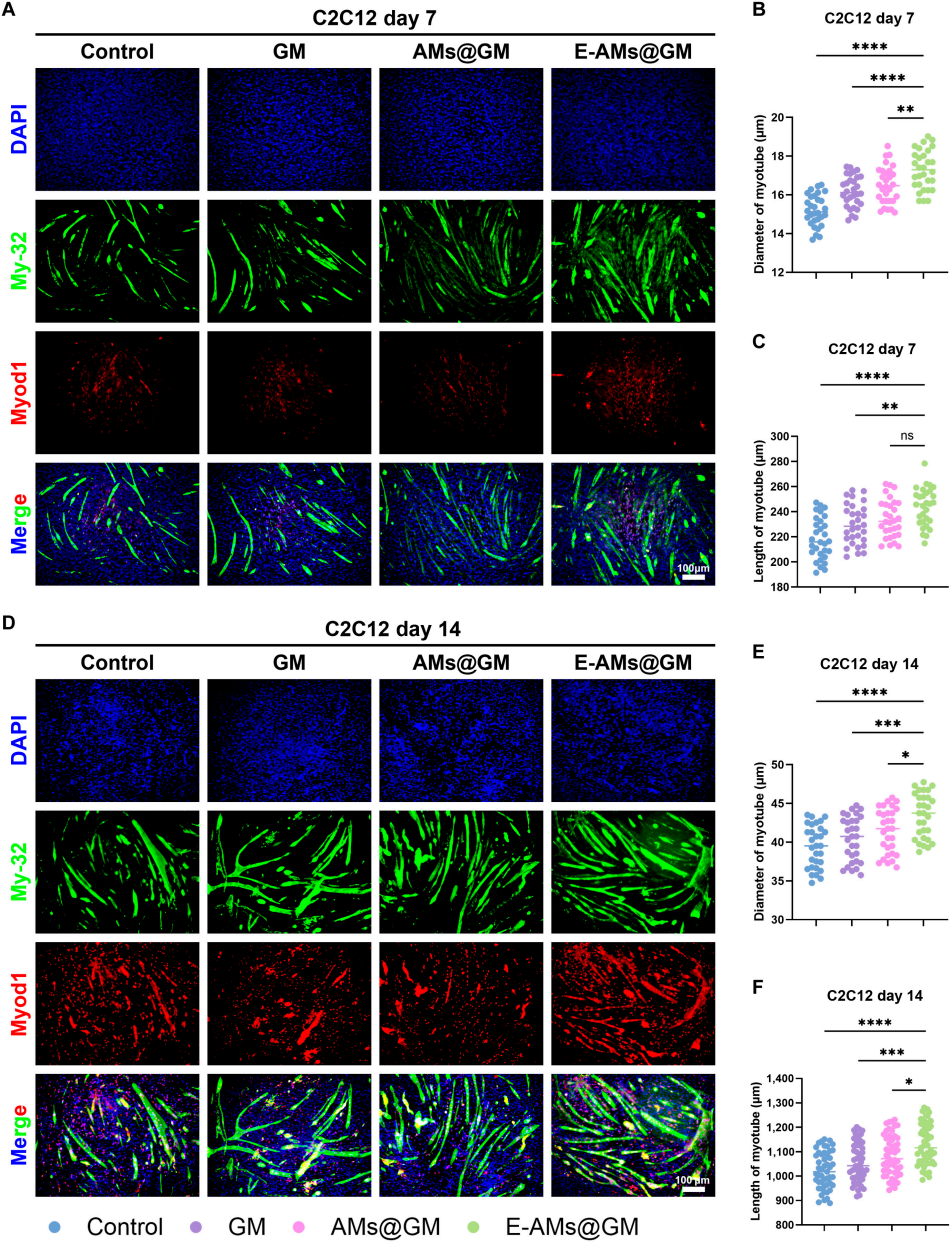
Effects of E-AMs@GM on C2C12 myogenic differentiation in vitro. C2C12 myoblasts were induced for myogenic differentiation and cultured under the indicated conditions (Control, GM, AMs@GM, and E-AMs@GM). (A) Representative immunofluorescence images at day 7 showing nuclei (DAPI, blue), My32/MyHC (green), and MyoD1 (red), with merged views. (B and C) Quantification of myotube diameter (B) and myotube length (C) at day 7. (D) Representative immunofluorescence images at day 14 stained for DAPI (blue), My32/MyHC (green), and MyoD1 (red), with merged views. (E and F) Quantification of myotube diameter (E) and myotube length (F) at day 14. Data are presented as mean ± SD (*n* = 5). Statistical significance was determined using one-way analysis of variance followed by Tukey’s post hoc test. ns, not significant; *P < 0.05, **P < 0.01, ***P < 0.001, ****P < 0.0001. Scale bar, 100 μm.

To determine whether the material supports myogenesis, we used C2C12 myoblasts and assessed differentiation at days 7 and 14. Immunofluorescence staining for Myosin Heavy Chain (MyHC/My32) and MyoD1 revealed more robust formation of multinucleated myotubes in the E-AMs@GM group than in controls (Fig. 4A and D). Morphometric quantification further demonstrated significantly increased myotube diameter and myotube length on E-AMs@GM at both time points (Fig. 4B, C, E, and F). These results suggest that the emodin-depot composite provides a bioactive microenvironment that facilitates myogenic commitment and maturation.

### E-AMs@GM promotes skeletal muscle regeneration and attenuates fibrosis in vivo

**Fig. 5.**
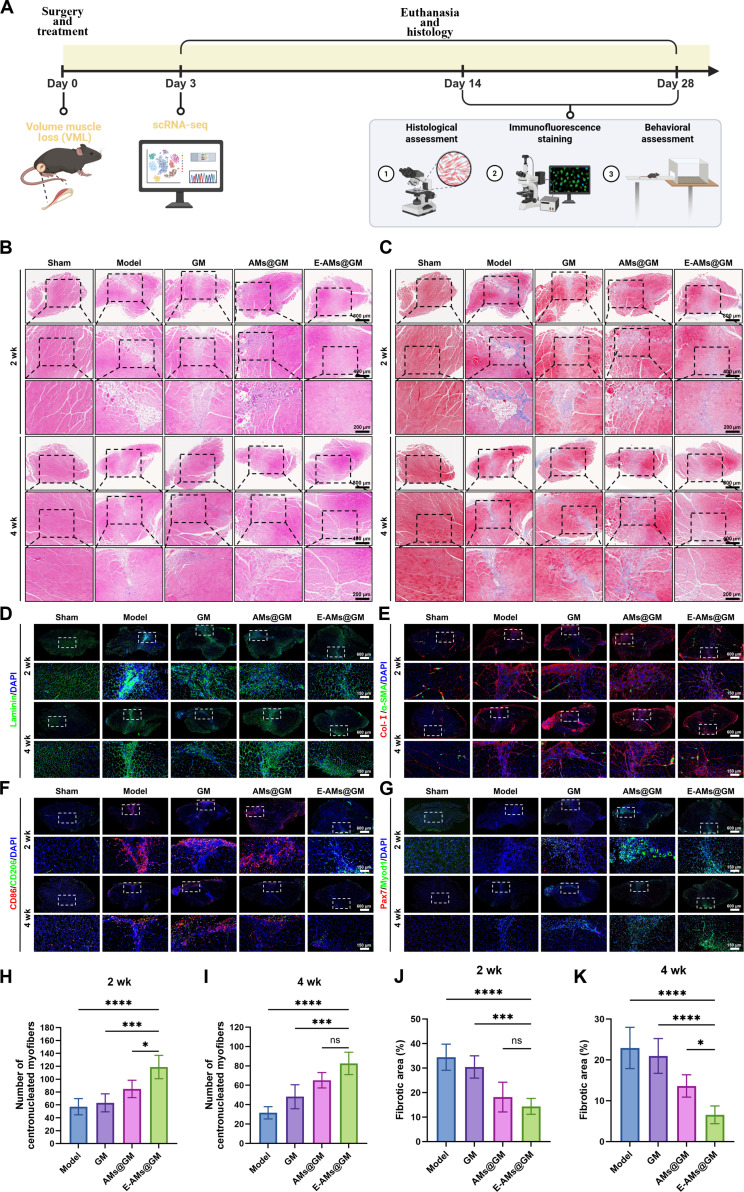
E-AMs@GM promotes structural regeneration and modulates the inflammatory–fibrotic–myogenic microenvironment in a murine volumetric muscle loss (VML) model. (A) Schematic overview of the in vivo study design, including establishment of the VML defect, implantation of the indicated hydrogels (GM, AMs@GM, or E-AMs@GM), and the scheduled time points for tissue harvest and analyses. (B and C) Representative histological images of the defect region at 2 and 4 wk post-implantation, respectively, showing hematoxylin and eosin staining for overall tissue architecture and cellular infiltration and Masson’s trichrome staining for collagen deposition and fibrosis. (D to G) Representative immunofluorescence images of the regenerated tissue at the indicated time points, stained for Laminin (myofiber basement membrane delineation), Collagen I/III and alpha-smooth muscle actin (α-SMA; fibrotic extracellular matrix deposition and myofibroblast activation), CD86 (M1-like pro-inflammatory macrophages), CD206 (M2-like pro-regenerative macrophages), and PAX7 with MyoD1 (satellite cell pool and myogenic activation), with nuclei counterstained by DAPI. (H to K) Quantitative analyses corresponding to the histological and immunofluorescence assessments, including indices of myofiber regeneration (laminin-defined myofiber metrics), fibrosis (collagen and α-SMA-positive area), macrophage polarization balance (CD86^+^ and CD206^+^ cells/area or fluorescence intensity), and myogenic progenitor activation (PAX7^+^ and MyoD1^+^ cell counts/area). Data are presented as mean ± SD (*n* = 5). ns, not significant; *P < 0.05, ***P < 0.001, ****P < 0.001. Scale bars are indicated in the images.

We next evaluated therapeutic efficacy in a murine VML model implanted with GM, AMs@GM, or E-AMs@GM. Histological analysis (hematoxylin and eosin and Masson’s trichrome) at 2 and 4 weeks showed that E-AMs@GM markedly improved tissue organization and reduced collagen-rich scar formation, whereas untreated/model and GM groups exhibited more pronounced fibrosis and atrophic remodeling (Fig. 5B and C).

Immunofluorescence further confirmed niche remodeling toward constructive regeneration. E-AMs@GM increased the density and continuity of Laminin-positive myofiber boundaries, while reducing Collagen I deposition and alpha-smooth muscle actin signals indicative of fibrotic ECM accumulation and myofibroblast activation (Fig. 5D and E). In parallel, macrophage profiling in situ showed a higher CD206^+^/CD86^+^ balance consistent with a pro-regenerative immune milieu (Fig. 5F). Importantly, E-AMs@GM also increased PAX7^+^ and MyoD1^+^ myogenic progenitor populations, suggesting enhanced activation/maintenance of endogenous satellite cells within the defect niche (Fig. 5G). Quantification of these histological and immunofluorescence indices corroborated the above trends (Fig. 5H to K).

### E-AMs@GM restores hindlimb locomotor function as assessed by CatWalk gait analysis

**Fig. 6.**
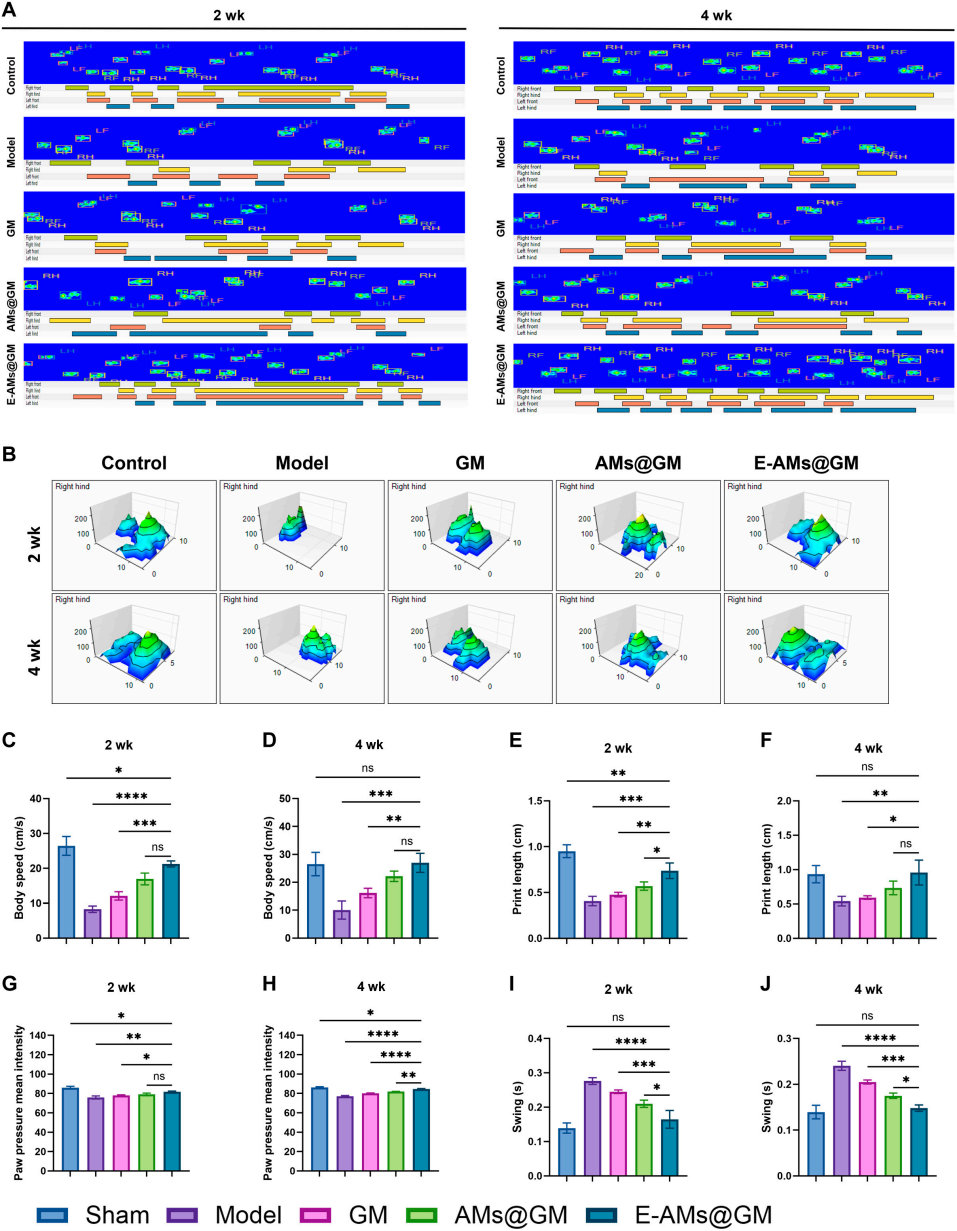
CatWalk-based gait analysis demonstrates functional restoration of injured hindlimb muscle following hydrogel treatment in the murine VML model. Comprehensive locomotor function was assessed using the CatWalk XT automated gait analysis system at the indicated postoperative time points for mice treated with GM, AMs@GM, or E-AMs@GM. (A) Representative CatWalk footprint recordings and paw-contact maps illustrating paw placement, contact area distribution, and dynamic load transfer during voluntary locomotion. (B) Representative force–time profiles/pressure curves of the affected hind paw showing peak contact/maximum intensity (i.e., peak load) and overall mechanical output during stance. (C to J) Quantitative gait parameters derived from CatWalk analysis, including metrics of paw-print morphology and intensity (e.g., print area, maximum contact area, and mean/maximum intensity), temporal parameters (e.g., stand time, swing time, and duty cycle), spatial parameters (e.g., stride length and base of support), and interlimb coordination indices (e.g., regularity index and/or phase dispersion), as indicated in each panel. Data are presented as mean ± SD (*n* = 5). ns, not significant; *P < 0.05, **P < 0.01, ***P < 0.001, ****P < 0.001.

To determine whether histological regeneration translated into functional recovery, we performed CatWalk XT gait analysis. Representative footprint maps and dynamic force/pressure profiles showed that VML injury severely impaired paw placement and weight-bearing mechanics, whereas E-AMs@GM treatment substantially normalized these patterns (Fig. 6A and B).

Across multiple CatWalk-derived parameters—including paw-print morphology and loading intensity (e.g., maximum contact area and pressure-related indices), as well as additional temporal/spatial gait metrics—E-AMs@GM consistently improved functional outcomes and approached sham-like performance (Fig. 6C to J). These findings confirm that E-AMs@GM not only improves tissue-level repair but also yields meaningful restoration of locomotor function.

### In vivo biosafety evaluation

Finally, systemic biosafety was assessed by histological examination of major organs (heart, liver, spleen, lung, and kidney). Hematoxylin and eosin and Masson’s trichrome staining revealed no overt inflammatory infiltration, tissue necrosis, or abnormal fibrosis in the E-AMs@GM group, indicating a favorable in vivo safety profile (Fig. S1).

### Single-cell transcriptome mapping revealed the cellular basis of E-AMs@GM remodeling the skeletal muscle repair microenvironment

**Fig. 7.**
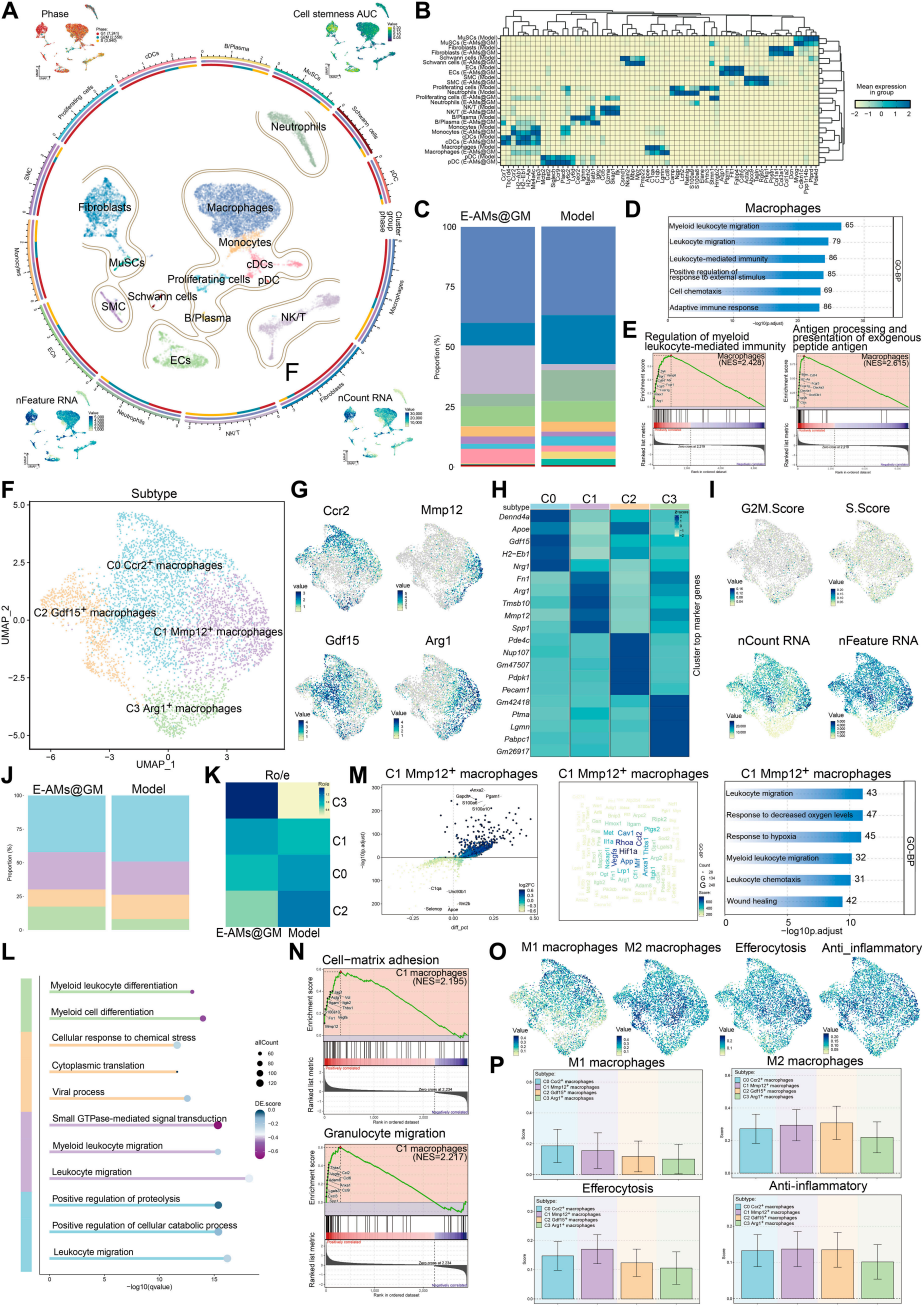
Visualization of single-cell microenvironment and macrophages in mouse skeletal muscle tissue after E-AMs@GM intervention. (A) The Uniform Manifold Approximation and Projection (UMAP) plots show the cell clustering within skeletal muscle tissue. Cells were colored according to their respective categories, and contour curves defined the boundaries of each cell type. Three concentric rings (from the outside in) represent the proportion of different clusters within each group, the sample source, and the cell cycle stage, respectively. The three concentric circles (from outer to inner) represent different clusters, groups, and cell-cycle phases, respectively. Starting clockwise from the top left corner, 4 UMAP plots sequentially show the expression distribution of phase, cell stemness score, nCount RNA score, and nFeature RNA score. AUC, area under the curve. (B) The heatmap shows the expression of Top5 differentially expressed genes (DEGs) for different cell types in the E-AMs@GM group and the model group. (C) The scale plots showed the proportion of different cell types in the mouse skeletal muscle injury repair microenvironment in the E-AMs@GM group and the model group. (D) The bar plot shows the Gene Ontology Biological Process (GO-BP) functional enrichment analysis results for macrophages. (E) Gene set enrichment analysis (GSEA) plots for macrophages are shown. These 2 biological processes exhibit a positive enrichment trend in macrophages: “Regulation of myeloid leukocyte-mediated immunity” and “Antigen processing and presentation of exogenous peptide antigen”. NES, normalized enrichment score. (F) The UMAP plot shows the cluster distribution of the 4 macrophage subtypes. (G) The UMAP plots show the distribution of characteristic genes of the 4 macrophage subtypes. (H) The heatmap shows the Top5 highly expressed DEGs in macrophages. (I) The UMAP plots show the distribution of nCount RNA, nFeature RNA, and cell-cycle-related scores (G2M.Score and S.Score) in the 4 macrophage subtypes. (J) The scale plots show the percentage of the 4 macrophage subtypes in the E-AMs@GM group and the model group. (K) The Ro/e preference analysis heatmap shows the preference distribution of the 4 macrophage subtypes in the model group and the E-AMs@GM group. (L) GO enrichment analysis shows the biological enrichment pathways of the 4 macrophage subtypes. (M) Left: DEGs volcano plot of the C1 *Mmp12^+^* macrophages; middle: word cloud plot of gene function enrichment for C1 *Mmp12^+^* macrophages; right: GO-BP functional enrichment analysis results for C1 *Mmp12^+^* macrophages. (N) GSEA plots for C1 *Mmp12^+^* macrophages are shown. These 2 biological processes exhibit a positive enrichment trend in C1 *Mmp12^+^* macrophages: “Cell-matrix adhesion” and “Granulocyte migration”. (O and P) The activity of M1-macrophage-related gene sets, M2-macrophage-related gene sets, endocytosis gene sets, and anti-inflammatory gene sets was evaluated across different macrophage subtypes.

To elucidate the transcriptomic characteristics of the skeletal muscle defect repair microenvironment in mice after intervention with the innovative material E-AMs@GM, we performed scRNA sequencing (scRNA-seq) on skeletal muscle samples from each mouse in the model group and the E-AMs@GM group. After normalizing the sequencing data, a total of 13,339 high-quality single cells were obtained for Uniform Manifold Approximation and Projection plot (Fig. 7A). By utilizing typical markers associated with cell populations and the expression levels of these differentially expressed genes in cells, we identified the major cell types as macrophages, fibroblasts, natural killer/T (NK/T) cells, neutrophils, endothelial cells, monocytes, smooth muscle cells, proliferating cells, conventional dendritic cells, B/Plasma cells, muscle satellite cells (MuSCs), Schwann cells, and plasmacytoid dendritic cells (Fig. S2A). NK/T cells were predominantly from the E-AMs@GM group, and macrophages were similarly distributed between the 2 groups. Macrophages were primarily in the G1 phase (Fig. S2B). Figure 7B shows the differences in gene expression changes for each cell type between the model group and the E-AMs@GM group. In the E-AMs@GM group, MuSCs highly expressed genes related to the transition from “proliferation state” to “differentiation state”, such as *Myog* and *Cdkn1c* [42,43]. In the E-AMs@GM group, macrophages highly express genes related to cell recruitment, clearing, and reconstruction, such as *Lgmn* and *Apoe* [44,45], preventing macrophages from over-inhibition and the risk of fibrosis [46]. The study found that, compared with the model group, the proportion of MuSCs decreased in the E-AMs@GM group, while the proportion of macrophages increased significantly. The ratio of observed to expected (Ro/e) heatmaps and bar plots confirmed that MuSCs were biased toward the model group and the G1 phase, while NK/T cells were biased toward the E-AMs@GM group and the S phase (Fig. 7C and Fig. S2C to E). Furthermore, enrichment analysis showed that macrophages were significantly enriched in immune response pathways such as “Regulation of myeloid leukocyte-mediated immunity” and “Antigen processing and presentation of exogenous peptide antigen”. Macrophages were highly enriched for *Cd300lb*, *Igsf6*, *Cd93*, *Ccl3*, and *Irf8*, suggesting a potential transition from M1 to M2 macrophages [47,48] (Fig. 7D and E and Figs. S2F). Network plot and Gene Ontology (GO) enrichment analysis supported the inferred biological functions of macrophages plot (Fig. S2G and H).

### The innovative material E-AMs@GM promoted muscle repair by enriching *Mmp12*^*+*^ anti-inflammatory macrophages

**Fig. 8.**
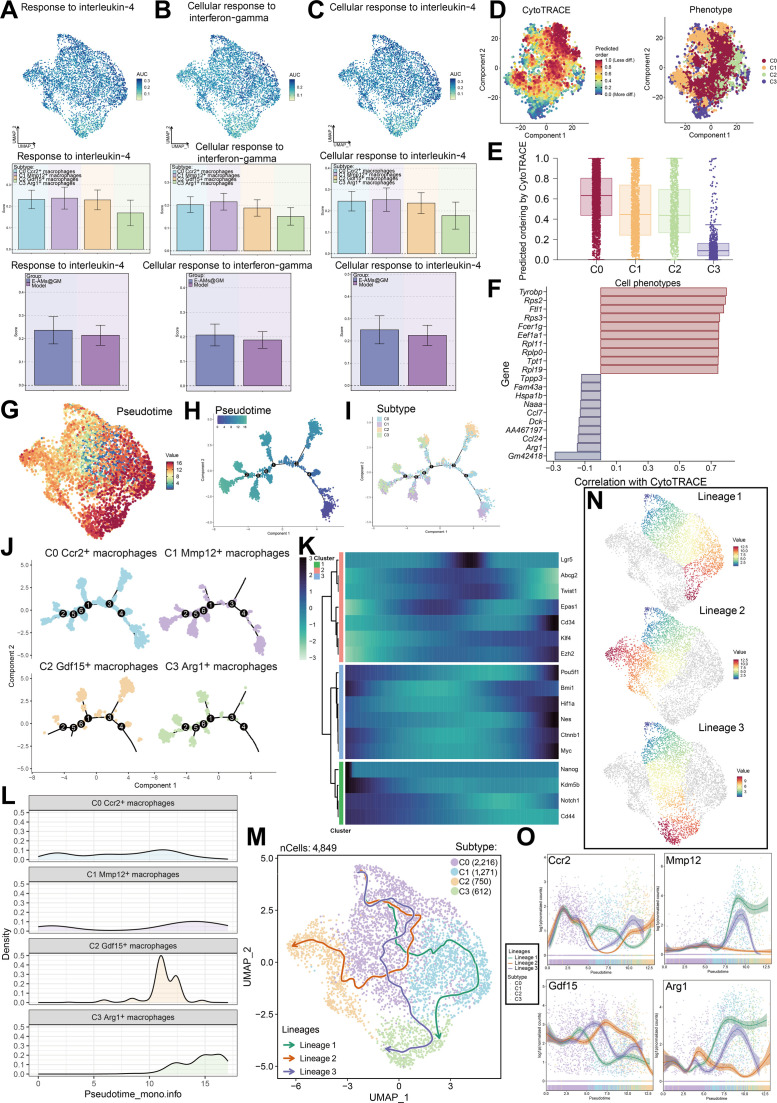
Stem changes and lineage evolution of macrophage subtypes driven by inflammatory factor responses. (A to C) The AUCell algorithm was used to calculate the activities of response to interleukin-4, cellular response to interferon-gamma, and cellular response to interleukin-4 in different macrophage subtypes and tissue origins. (D) The left plot shows the predicted ordering distribution of macrophages inferred by CytoTRACE, with colors representing differentiation potential; the right plot shows the CytoTRACE results for different macrophage subtypes, with colors representing different cell subtypes. (E) The box plot shows the stemness levels of the 4 macrophage subtypes based on CytoTRACE. (F) The bar chart shows the correlation analysis between stem genes and CytoTRACE. (G) The UMAP plot shows the visualization results of macrophage pseudotime generated by Monocle analysis. (H to J) Monocle analysis inferred the developmental trajectories of macrophages. Cells were colored by pseudotime, showing the distribution and dynamic differentiation trends of macrophage subtypes along the trajectories. (K) Based on pseudotime trends, a heatmap was used to visualize the clustering of differentially expressed genes in macrophages. (L) Density distribution of macrophage subtypes along pseudotime. Curves showed the distribution of C0 to C3 macrophages, reflecting dynamic changes during differentiation. (M and N) Differentiation trajectories of macrophages inferred by the Slingshot algorithm. Three lineages (Lineage 1, Lineage 2, and Lineage 3) are shown on the UMAP embedding. Solid curves represent the inferred developmental trajectories, and arrows indicate the direction of differentiation. (O) Dynamic expression patterns of *Ccr2*, *Mmp12*, *Gdf15*, and *Arg1* along pseudotime in different differentiation lineages. Cells are colored by macrophage subtypes, and the curves represent lineage-specific expression trends.

Given the importance of macrophages in skeletal muscle repair, we re-clustered macrophage subtypes to distinguish 4 subtypes and examined the expression patterns of their respective marker genes (*Ccr2*, *Mmp12*, *Gdf15*, and *Arg1*) [49] (Figs. 7F and G and Fig. S2L). We identified the differentially expressed genes distinguishing each macrophage subtype (Fig. 7H) and calculated the G2M.Score, S.Score, nCount RNA, and nFeature RNA for macrophages (Fig. 7I). C1 *Mmp12^+^* macrophages and C3 *Arg1^+^* macrophages were primarily from the E-AMs@GM group, while C0 *Ccr2^+^* macrophages and C2 *Gdf15^+^* macrophages were primarily from the model group (Fig. 7J and K and Fig. S2I). C1 *Mmp12^+^* macrophages showed a more even distribution across cell stages compared to the other 3 subtypes (Fig. S2J and K). It was worth noting that *Mmp12* belongs to the matrix metalloproteinase family and was a zinc-dependent endopeptidase found in the cytoplasm, nucleus, and phagolysosomes. In the nucleus, it played a role in transcriptional regulation, inhibiting the expression of genes such as proteasome activator complex subunit 3 and Secreted protein acidic and rich in cysteine-like protein 1 and binding to unidentified receptors on the cell and nuclear membranes to achieve intracellular translocation. Simultaneously, in macrophages, it exerted anti-inflammatory and anti-infective functions through both protease-dependent and protease-independent mechanisms [50]. *Mmp12* expression was closely related to inflammatory signaling pathways and matrix remodeling [51], and it could enhance the ability of keratinocytes to sense mechanical stress, geometric constraints, and matrix stiffness, thereby promoting cell migration and re-epithelialization [52]. Differential gene analysis and enrichment analysis of different macrophage subtypes revealed that C0 *Ccr2^+^* macrophages were mainly involved in functions related to immune defense, inflammatory response, and phagocytosis; C2 *Gdf15^+^* macrophages were involved in functions related to myeloid differentiation, guanosine triphosphatase signaling regulation, endocytosis, and stress transcription; and C3 *Arg1^+^* macrophages were involved in RNA splicing and processing, and protein localization. C1 *Mmp12^+^* macrophages, on the other hand, were involved in immune cell chemotaxis and migration, hypoxia and stress response, and tissue repair and ECM interaction. C1 *Mmp12^+^* macrophages might serve as a hub connecting “inflammatory clearance” and “tissue reconstruction”, marking a “turning point” in phenotypic transformation (Fig. 7L to N and Fig. S3A to E). Previous studies had shown that *Mmp12* not only participated in inflammation-related matrix remodeling but also played a regulatory role in the conversion of macrophages from pro-inflammatory M1 phenotype to anti-inflammatory M2 phenotype [53,54], and the C1 *Mmp12^+^* macrophages were suggested to be a key phenotypic mediator connecting the inflammation clearance and tissue repair phases. It was worth noting that macrophages exhibited remarkable plasticity, undergoing phenotypic polarization in response to cytokine stimuli, which was closely related to the local environment. Subsequently, we used the AUCell tool to assess the activity levels of M1- and M2-related genes in macrophage subtypes exhibiting M1-like or M2-like phenotypic characteristics during muscle inflammation. As shown in Fig. 7O and P, C1 *Mmp12^+^* macrophages showed enhanced gene activity associated with the M2 phenotype and scored highest in efferocytosis and anti-inflammatory functions. These findings were consistent with previous assumptions (Fig. S3F and G) [55], providing evidence that E-AMs@GM may reduce chronic inflammation in wounds and accelerate muscle repair. To verify this inference, we evaluated inflammation-related pathways in different macrophage subtypes and groups. Encouragingly, both C1 *Mmp12*^+^ macrophages and the E-AMs@GM group showed a trend toward relatively strong involvement in anti-inflammatory-related pathways, including “response to interleukin-4”, “cellular response to interferon-gamma”, and “cellular response to interleukin-4” (Fig. 8A to C).

### Dynamic polarization trajectories of *Mmp12^+^* macrophages and their role as a communication hub in tissue repair

In the early stages of inflammation, macrophages polarized into the pro-inflammatory M1 phenotype to clear pathogens and initiate an immune response. However, in the later stages of inflammation, as pathogens decreased and the need for repair increased, macrophages tended to polarize into the anti-inflammatory M2 phenotype, thereby promoting tissue repair and wound healing and suppressing persistent inflammation [56,57]. CytoTRACE analysis showed that C1 *Mmp12^+^* macrophages exhibited high stem cell characteristics (Fig. 8D to F). Subsequently, we used Monocle analysis to infer the possible trajectories of macrophage differentiation. Notably, as shown in Fig. 8G to K, C1 *Mmp12^+^* macrophages appeared in the early stages of the developmental trajectory and persisted throughout. Consistent with these findings, the expression level of the gene *Mmp12* increased at the beginning and near the end of the developmental trajectory (Fig. 8L). To further visualize the developmental trajectory and its potential differentiation direction, we applied the Slingshot algorithm to perform pseudotemporal analysis on 4 macrophage subtypes (Fig. 8M). The results showed that among the 3 main developmental lineages (Lineage 1, Lineage 2, and Lineage 3), C1 *Mmp12^+^* macrophages occupied an intermediate position in the pseudotime sequence, suggesting a possible transitional phenotype from an early stage to a terminal differentiation stage (Fig. 8N). Furthermore, analysis of gene dynamics revealed that *Mmp12* showed an overall upward trend over time in Lineage 1 (Fig. 8O). Next, we used the single-cell regulatory network inference and clustering (SCENIC) identification rules and the connection specificity index (CSI) matrix to reveal the regulatory modules among macrophage subtypes. Based on the AUCell scores, we divided these regulatory modules into 4 regions, M1 to M4 (Fig. S4A). Subsequently, we ranked the transcription factor (TF) activity scores in M1 to M4 within each subtype. The scatter plot showed that the C1 *Mmp12^+^* macrophage score was higher in M4. In M4, the E-AMs@GM group scored higher than the model group (Fig. S4B and C). We also ranked the transcription factors in M4 according to the variance explained across subtypes (Fig. S4D). To understand the differences in transcriptional TFs among the subtypes, we attempted to visualize their transcriptional regulatory patterns. First, we showed the Top5 TFs in each subtype, where the Top5 TFs for C1 *Mmp12^+^* macrophages were *Ets2*, *Fosl2*, *Hif1a*, *Cebpb*, and *Churc1* (Fig. S4E and F). Next, we used the AUCell algorithm to score the regulatory factors of C1 *Mmp12^+^* macrophages. The UMAP plots showed that *Ets2*, *Fosl2*, *Hif1a*, *Cebpb*, and *Churc1* all exhibit higher expression levels in C1 *Mmp12^+^* macrophages (Fig. S4G), while the bar plot provided a direct comparison (Fig. S4H and I).

**Fig. 9.**
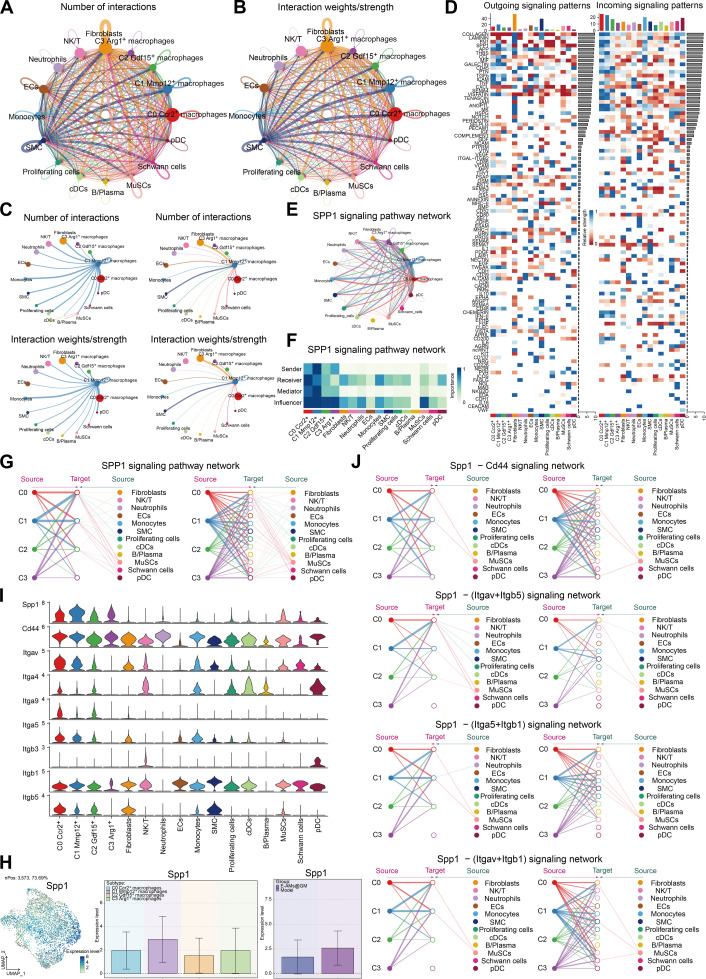
Analysis of the macrophages cell communication network and characteristics of cell–cell interactions mediated by the SPP1 signaling pathway. (A) The circular diagram shows the overall structure of the global communication network, illustrating the quantity of interactions between macrophages and all other cell types. (B) The circular diagram shows the overall structure of the global communication network, illustrating the intensity of interactions between macrophages and all other cell types. (C) The circular plots show the cell interaction network of C1 *Mmp12^+^* macrophages as source and target, with the left side representing the source and the right side representing the target. They were arranged from top to bottom in order of quantity and intensity. (D) The heatmaps show the outgoing and incoming signaling patterns between the 4 macrophage subtypes and other cell types. (E) The circular plot shows the interaction network of macrophage subtypes with other cell types in the SPP1 signaling pathway. (F) The heatmap shows the communication centrality scores of different cell types as senders, receivers, mediators, and influencers in the SPP1 signaling pathway. (G) The hierarchical diagram shows the interactions between macrophage subtypes (C0 to C3) and other cell types within the SPP1 signaling pathway. (H) UMAP and bar charts show the expression of *Spp1* in different macrophage subtypes and cell sample sources. (I) Violin plots illustrate the expression levels of the ligand Spp1 and its relevant receptors (*Cd44*, *Itgav*, *Itga4*, *Itga9*, *Itga5*, *Itgb3*, *Itgb1*, and *Itgb5*) across macrophage subtypes (C0 to C3) and various other cell types. (J) Hierarchical plots show the inferred intercellular communication strength of 4 specific ligand–receptor pairs (Spp1–Cd44, Spp1–Itgav+Itgb5, Spp1–Itga5+Itgb1, and Spp1–Itgav+Itgb1) between macrophage subtypes (C0 to C3) and other cell types.

To elucidate the intercellular communication mechanism between C1 *Mmp12^+^* macrophages and other cells in the tissue microenvironment, we performed cell communication analysis (Fig. 9A and B). The interactions between macrophage subtypes and various cell types were quite prominent. To illustrate this visually, we used a circle plot to show the number and intensity of interactions between C1 *Mmp12^+^* macrophages and other cell types. The results showed that there was an interaction between C1 *Mmp12^+^* macrophages and MuSCs (Fig. 9C). Next, we specifically demonstrated the afferent communication patterns and the efferent communication patterns, aiming to quantify the quantity, intensity, and incoming/outgoing signals of all intercellular interactions. We could see that in outgoing signaling patterns, C1 *Mmp12^+^* macrophages were prominently expressed in pathways such as SPP1, APP, MIF, and TNF; while in incoming signaling patterns, C1 *Mmp12^+^* macrophages were prominently expressed in pathways such as FN1, MK, THBS, and THY1 (Fig. 9D). Furthermore, subsequent analysis of hierarchical clustering suggested that this may have been due to a potential association between C1 *Mmp12^+^* macrophages and the SPP1 signaling pathway (Fig. 9E). Analysis of the SPP1 signaling pathway network centrality scores showed that in these cell populations, C1 *Mmp12*^+^ macrophages acted as sender, mediator, and influencer, while MuSCs acted as influencer and receiver. C1 *Mmp12*^*+*^ macrophages were predicted to interact with MuSCs based on ligand–receptor inference. These analyses suggest that C1 *Mmp12*^+^ macrophages may serve as predominant signal emitters in the SPP1 signaling pathway, while MuSCs appear to function as primary receivers, though direct experimental validation of this axis remains to be established (Fig. 9F). Notably, C1 *Mmp12*^*+*^ macrophages may engage in paracrine interactions with MuSCs within the SPP1 signaling pathway, thereby facilitating substantial communication between these cell populations (Fig. 9G). Meanwhile, we showed the expression distribution and expression status of the receptors and ligands, further confirming the significant presence of *Spp1* as a ligand (Fig. 9H and Figs. S4K and L). To identify other key receptor proteins, we used circle plots, hierarchical clustering plots, and violin plots to illustrate the interactions between C1 *Mmp12^+^* macrophages and other cell types. In the SPP1 signaling pathway, Spp1-(ltgav+ltgb1), Spp1-(ltga5+ltgb1), Spp1-(ltgav+ltgb5), and Spp1-Cd44 were the major ligands and receptors for signal transduction in these 2 cell types (Fig. 9I and Fig. S4J). Paracrine signals were present in the Spp1-Cd44 signaling network, Spp1-(Itgav+Itgb5) signaling network, Spp1-(Itga5+Itgb1) signaling network, and Spp1-(Itgav+ltgb1) signaling network, suggesting that they might have promoted skeletal muscle repair by coordinating macrophages and MuSCs (Fig. 9J). These results further supported the findings of scRNA-seq, indicating that E-AMs@GM were linked to MuSCs in accelerating muscle damage repair by promoting the aggregation of M2-phenotype macrophages and reducing levels of inflammatory stimulation [58].

## Discussion

VML represents one of the most recalcitrant forms of musculoskeletal injury because the defect is not simply a loss of volume but a collapse of the regenerative niche: The myofiber template, satellite-cell microenvironment, vascular support, and immune homeostasis are simultaneously disrupted. Clinically, current reconstructive approaches—such as autologous muscle transfer, free functional flaps, and staged debridement—can restore coverage yet frequently fall short in recreating contractile architecture and long-term function, with fibrosis and chronic inflammation remaining dominant endpoints. Against this background, biomaterials for VML have increasingly shifted from “space fillers” to “instructive scaffolds”, aiming to actively guide immune resolution and myogenesis. In this study, we present an injectable, photocurable emodin-depot microsphere-in-hydrogel platform (E-AMs@GM) that integrates structural support (GelMA network) with spatiotemporally sustained immunomodulation (emodin-loaded alginate microspheres). Across material characterization, in vitro assays, and a murine VML model, E-AMs@GM achieved coordinated improvements in inflammation control, myogenic regeneration, fibrosis suppression, and gait-level functional recovery. Importantly, single-cell analysis further indicated that these benefits are coupled to macrophage state remodeling and an SPP1-centered communication axis, providing a mechanistic link between material design and niche reprogramming.

A large body of VML biomaterials research has focused on restoring a permissive physical substrate using decellularized ECM scaffolds, collagen/gelatin matrices, fibrin constructs, and synthetic polymer scaffolds [1,4,8]. These approaches provide topographical guidance and cell adhesion cues, yet their outcomes are often limited by the persistence of a pro-inflammatory wound environment and the rapid dominance of fibrotic remodeling. In many studies, scaffolds improve histological organization but yield incomplete functional recovery, underscoring that mechanics and architecture alone are insufficient when the immune niche remains maladaptively activated [3–6].

Parallel lines of work have therefore attempted skeletal muscle repair by coordinating macrophages and MuSCsto add “bioactivity” to VML scaffolds, primarily through growth factors, including insulin-like growth factor 1, vascular endothelial growth factor, and hepatocyte growth factor, chemokines, or exogenous cell delivery (MuSCs and MSCs) [59,60]. While such strategies can enhance angiogenesis or myogenesis, they face recurring practical barriers: burst release and short half-life for proteins, high cost and cold-chain constraints, and variability or regulatory complexity for cell-based products [61–64]. Moreover, growth factor supplementation does not necessarily resolve the underlying immune dysregulation; in some settings, it can even exacerbate aberrant remodeling if the inflammatory phase is not properly controlled [16,65,66]. These limitations have fueled interest in immunoengineering approaches that target the early inflammatory cascade and macrophage programs, which are increasingly recognized as upstream “master regulators” of regeneration versus fibrosis.

Within this evolving landscape, E-AMs@GM offers a deliberately immune-first regenerative logic. Rather than relying primarily on exogenous cells or labile protein factors, our platform uses a small-molecule depot to deliver sustained immunoregulatory cues while the GelMA matrix provides a cell-adhesive structural framework [20,23,24]. This design directly addresses several recurrent issues in the literature: (a) ensuring durable local bioavailability without repeated dosing, (b) preserving injectability and defect conformity, and (c) synchronizing bioactivity with the temporal dynamics of VML healing. GelMA has been widely used in tissue engineering because it is photocrosslinkable, cytocompatible, and contains native cell-adhesive motifs [21,25]. However, GelMA alone is typically a permissive substrate rather than a strongly instructive immunoregulatory scaffold; in hostile injury contexts, its regenerative efficacy can be limited without additional niche-shaping signals. Alginate microspheres, conversely, are a robust depot format for controlling diffusion and protecting encapsulated cargo [67–69], yet alginate by itself is relatively bioinert and does not provide the adhesion-rich framework needed for myogenic progression.

By embedding alginate microspheres within GelMA, E-AMs@GM leverages complementary advantages that are frequently separated across prior studies: a tissue-like adhesive scaffold and a spatially distributed sustained-release module. Compared with direct drug mixing into a hydrogel (often leading to burst release and rapid loss), the microsphere depot architecture is expected to smooth release kinetics and maintain bioactivity throughout the early inflammatory window—precisely when macrophage programming and fibrotic commitment are most sensitive [70–73]. In addition, our macroscopic and interfacial observations suggest a practical benefit that is underemphasized in the literature: retention and adherence at the implant–tissue interface. In dynamic tissues such as skeletal muscle, poor interfacial stability can undermine otherwise effective biomaterials [17,74,75]. Therefore, the combination of defect conformity, depot-mediated release control, and adhesion may represent a meaningful engineering package for VML deployment.

Small molecules have been less explored than proteins or cells in VML biomaterials, despite offering clear translational advantages: chemical stability, scalable manufacturing, and more straightforward quality control [76–78]. Emodin is particularly attractive because its reported anti-inflammatory and antioxidant activities are mechanistically aligned with VML pathophysiology, where excessive cytokine signaling and ROS contribute to impaired myogenesis and fibrotic conversion [79–81]. Nevertheless, free small-molecule administration is typically hampered by low aqueous solubility, rapid diffusion, and systemic exposure [76,82,83]. Our results support the premise that a depot-enabled local delivery format can unlock small-molecule potential in VML by maintaining a therapeutic microenvironment while limiting off-target dilution.

Consistent with this rationale, E-AMs@GM showed cytocompatibility and enhanced migration of reparative cells in vitro, while reducing oxidative-stress signals under challenge conditions. These findings fit well with a broader regenerative biomaterials trend: Protecting cell competence and reducing stress signaling early can be a prerequisite for successful downstream differentiation and tissue assembly [84,85]. In the context of VML, this is particularly relevant because oxidative/inflammatory stress not only damages cells but also biases the defect toward fibro-inflammatory remodeling. The importance of macrophage programs in muscle repair has been repeatedly demonstrated across injury models: Inflammatory macrophage activity is necessary for debris clearance, yet prolonged or excessive inflammatory signaling suppresses myogenic differentiation and promotes fibrotic remodeling. Many biomaterial studies report changes in “M1/M2 markers”, but mechanistic interpretation is often limited by the oversimplification of macrophage biology and by the lack of cell-state resolution [86–88]. A key contribution of the present study is that we combined conventional polarization readouts (CD86/CD206, cytokines, and transcriptional markers) with single-cell mapping, enabling a more nuanced view of macrophage state composition and signaling behavior in the VML niche. It should be noted that the use of CD86 and CD206 as M1- and M2-associated markers in the conventional immunofluorescence and gene expression assays reflects standard practice for cross-study comparability in the VML biomaterials field and does not imply endorsement of a strict M1/M2 binary. Macrophage activation states are now well recognized to exist along a continuous and context-dependent spectrum. The single-cell transcriptomic analysis presented here explicitly addresses this complexity by identifying 4 transcriptionally distinct macrophage subtypes with functional characteristics that extend well beyond the classical M1/M2 dichotomy, providing a more granular mechanistic framework for interpreting the immunomodulatory effects of E-AMs@GM.

Our in vitro and in vivo data converge on the conclusion that E-AMs@GM shifts macrophage programs toward pro-regenerative phenotypes, consistent with reduced inflammatory cytokine output and reduced fibrotic marker expression in tissue. Beyond the histological and functional improvements, the updated single-cell atlas provides a mechanistic framework for how E-AMs@GM reprograms the VML repair microenvironment. scRNA-seq profiling generated a high-quality map of 13,339 cells, revealing coordinated shifts in both immune and myogenic compartments. At the population level, E-AMs@GM was associated with a relative increase in macrophage representation alongside a reduction in MuSC proportion, yet MuSCs in the E-AMs@GM condition displayed transcriptional features consistent with progression from proliferation toward differentiation, exemplified by elevated Myog and Cdkn1c. In parallel, macrophages in the E-AMs@GM group up-regulated genes linked to cell recruitment, clearance, and tissue reconstruction (e.g., *Lgmn* and *Apoe*), supporting a state that favors resolution rather than persistent inflammatory stalling. Enrichment analyses further indicated that macrophages were preferentially engaged in immune programs such as regulation of myeloid leukocyte-mediated immunity and antigen processing/presentation, consistent with active immune orchestration during the transition from early inflammation to regenerative remodeling.

A key advance in the updated analysis is the identification of a repair-associated macrophage subset enriched by E-AMs@GM. Macrophages were re-clustered into 4 subtypes (C0 Ccr2^+^, C1 Mmp12^+^, C2 Gdf15^+^, and C3 Arg1^+^), with C1 Mmp12^+^ and C3 Arg1^+^ macrophages predominantly originating from the E-AMs@GM group, whereas C0 Ccr2^+^ and C2 Gdf15^+^ macrophages were biased toward the model condition. Functionally, subtype-resolved differential expression and enrichment analyses suggested that C0 Ccr2^+^ macrophages were coupled to immune defense and inflammatory responses, while C1 Mmp12^+^ macrophages were preferentially linked to chemotaxis/migration, hypoxia–stress adaptation, tissue repair, and ECM interaction, consistent with a phenotype positioned at the interface of inflammatory clearance and reconstruction. Given that MMP12 is a matrix metalloproteinase with established roles in matrix remodeling and macrophage functional programs, its enrichment in the E-AMs@GM group supports a model in which the material does not merely suppress inflammation globally but rather selectively favors macrophage states that facilitate resolution and regenerative remodeling.

Macrophage plasticity is central to productive skeletal muscle regeneration, and the updated trajectory analyses strengthen the concept that C1 Mmp12^+^ macrophages act as a transitional hub. Gene-set activity scoring (AUCell) indicated that C1 Mmp12^+^ macrophages exhibited higher M2-associated activity, with prominent signatures related to efferocytosis and anti-inflammatory functions, consistent with a pro-resolving phenotype. CytoTRACE and pseudotime inference further placed C1 Mmp12^+^ macrophages in earlier and intermediate positions along developmental trajectories, persisting across stages rather than appearing only as a terminal state. Slingshot-based lineage modeling suggested that C1 Mmp12^+^ macrophages occupy an intermediate pseudotime position and may represent a phenotypic “turning point” connecting early inflammatory programs to later repair-associated endpoints, while gene dynamics supported sustained or re-emergent Mmp12 activity along dominant differentiation paths.

The updated regulatory and communication analyses provide additional mechanistic specificity. Regulatory network inference (SCENIC/CSI-based modules) highlighted a macrophage regulatory program enriched in the E-AMs@GM group, with C1 Mmp12^+^ macrophages showing elevated activity in a repair-associated module (M4). Among the top transcription factors associated with C1 Mmp12^+^ macrophages, Ets2, Fosl2, Hif1a, Cebpb, and Churc1 emerged as candidate regulators of this state, consistent with integration of inflammatory resolution, stress adaptation, and reparative remodeling signals.

Critically, cell–cell communication analysis identified SPP1 signaling as a prominent axis connecting C1 Mmp12^+^ macrophages with MuSCs. Network centrality suggested that C1 Mmp12^+^ macrophages act as major senders/mediators of SPP1 signals, whereas MuSCs function predominantly as receivers/influencers, implying directional macrophage-to-MuSC instruction. Ligand–receptor mapping further supported substantive paracrine interactions through SPP1–CD44 and SPP1–integrin pairs, including SPP1–(ITGAV+ITGB1), SPP1–(ITGA5+ITGB1), and SPP1–(ITGAV+ITGB5). Together, these data refine the mechanistic model: E-AMs@GM promotes accumulation of pro-resolving macrophage states (notably Mmp12^+^), which may engage MuSCs through SPP1-centered communication to potentially coordinate ECM remodeling and myogenic progression, consistent with accelerated transition from inflammatory stagnation to functional muscle regeneration.

A persistent critique in the VML biomaterials field is the disconnect between structural improvements and functional recovery [1,4,9]. Many scaffolds improve histology yet yield limited restoration of strength or locomotion because newly formed tissue lacks adequate maturation, organization, or integration [1,8,9]. In our study, the improvements in tissue architecture and reduced fibrosis were accompanied by CatWalk-based gait recovery, suggesting that E-AMs@GM does not merely alter staining patterns but meaningfully improves limb biomechanics. This functional outcome likely reflects 2 coordinated effects: (a) reduced fibrotic stiffening and chronic inflammation, which can impair contractility and force transmission; and (b) improved myogenic progression, consistent with our in vitro differentiation results and in vivo satellite-cell activation signatures. More broadly, these findings reinforce a theme seen across recent high-performing VML biomaterial studies: Immunomodulation and myogenesis must be coupled, and successful materials are those that synchronize the early immune transition with later-stage tissue assembly.

This study is limited by the inherent constraints of a murine model and by the correlative nature of ligand–receptor inference in single-cell analysis. Additionally, the in vitro myogenic differentiation experiments were conducted using C2C12 cells, an immortalized myoblast line that may not fully recapitulate the behavior of primary muscle satellite cells in vivo; future studies using primary satellite cells or ex vivo muscle fiber cultures would provide additional physiological relevance to these findings. Future work will also focus on validating causality of key signaling nodes (e.g., perturbation of SPP1–receptor interactions), incorporating direct muscle force measurements, and extending evaluation to longer-term maturation and more clinically representative models.

Overall, by combining a depot-enabled small-molecule immunomodulator with a photocurable, defect-conforming scaffold, and by linking functional recovery to single-cell-resolved niche remodeling, this work advances an emerging paradigm for VML repair: materials that instruct immune–myogenic ecosystems rather than simply replace lost volume.

## Conclusion

In this work, we developed an injectable and photocurable emodin-depot microsphere-in-hydrogel platform (E-AMs@GM) that integrates a GelMA structural matrix with alginate-microsphere-mediated sustained delivery of emodin. E-AMs@GM demonstrated favorable physicochemical properties and biosafety, promoted myogenic differentiation, and reshaped macrophage programs toward a pro-regenerative phenotype in vitro. In a murine VML model, E-AMs@GM reduced inflammation and fibrosis, enhanced muscle regeneration, and improved locomotor function. Single-cell profiling further suggested that therapeutic benefits are linked to macrophage-state remodeling and SPP1-centered niche communication. Collectively, these findings support E-AMs@GM as an instructive biomaterial strategy for volumetric muscle repair and provide a mechanistic basis for future translational development.

## Materials and Methods

The detailed methodology can be found in the supplementary file.

## Ethical Approval

All animal procedures were conducted as per “The Guide for Care and Use of Laboratory Animals”, aiming to minimize animal suffering, and were approved by the Guangxi Medical University Institutional Animal Care and Use Committee (Approval No. 2023.06266).

## Data Availability

All data are available in the main text or the supplementary materials. The materials generated are available from the lead contact upon reasonable request.
