# Supplementary material for: An Emodin-Depot Microsphere-in-Hydrogel Reprograms the Immuno-myogenic Niche to Enable Volumetric Muscle Loss Repair Revealed by Single-Cell Profiling
Source: Research (Wash D C). 2026 Aug 12;9:1329. doi: 10.34133/research.1329 (PMC13462246; doi:10.34133/research.1329)
Supplement: Supplementary file 1 [file research.1329.f1.docx]

**Supporting information**

**An Emodin-Depot Microsphere-in-Hydrogel Reprograms the Immuno-Myogenic Niche to Enable Volumetric Muscle Loss Repair Revealed by Single-Cell Profiling**

**Authors:** *Wanshun Liu^1,#^,* *Ruizhe Wang^2,#^, Fu Zhao^3,#^, Zhengyuan Fang^4,#^, Jun Ma*^5^, *Zhixuan Mai^6^, Yanwei He^7^, Junzhe Sheng^2^, Yunxuan Shi^8^, Zhijie Zhao^9^, Hongling Jia^8^, Xiaojing Wang^10^, Wei Luo^6,7^, Renwen Wan^6,7^, Shiyi Chen^6,7^, Gang Chen^5,*^, Qi Sun^8,*^, Zhiwen Luo^5,6,7,11,12*^,* *Xinming Ye^11,*^, Nirong Bao^12,*^, Xiaochuan Gu^13 ,*^*

**2. Materials and Methods**

**2.1 Fabrication of alginate microspheres and emodin-loaded microspheres**

Sodium alginate microspheres (AMs) and emodin-loaded alginate microspheres (E-AMs) were fabricated via electrostatic spraying followed by ionic crosslinking. Briefly, alginate precursor (with or without emodin) was delivered through a syringe/needle under a high-voltage field and sprayed into a calcium chloride (CaCl₂) collecting bath to trigger rapid gelation and particle solidification. The resulting microspheres were collected, thoroughly washed to remove residual Ca²⁺ and unencapsulated molecules, and lyophilized for subsequent use. The fabrication workflow is schematized in Figure 1A.

**2.2 Preparation of microsphere-in-hydrogel composites (GM, AMs@GM, and E-AMs@GM)**

Gelatin methacryloyl (GelMA, 10% w/v) precursor solution was prepared in phosphate-buffered saline (PBS) and mixed with a photoinitiator (LAP, 0.25% w/v). For composite hydrogels, lyophilized AMs or E-AMs were dispersed into the GelMA/LAP precursor to obtain homogeneous suspensions. The mixtures were then cast into molds and photocrosslinked *in situ* using ultraviolet (UV) light (365 nm, 10 mW/cm²) for 60 seconds to form stable hydrogels. GelMA alone was defined as GM, blank microspheres embedded in GelMA as AMs@GM, and emodin-loaded microspheres embedded in GelMA as E-AMs@GM. Homogeneous distribution and stable entrapment of microspheres within the network were confirmed macroscopically (Figure 1B).

**2.3 Macroscopic shape conformability**

To evaluate defect-adaptive handling, the photocurable formulations were shaped into predefined geometries and crosslinked to assess shape fidelity and moldability. Representative demonstrations are shown in Figure 1C.

**2.4 Chemical characterization of GelMA functionalization**

Successful methacrylation of gelatin was verified using ¹H nuclear magnetic resonance (¹H NMR). The characteristic vinyl proton signals (relative to gelatin backbone peaks) were used to confirm GelMA functionalization (Figure 1D).

**2.5 Microstructure characterization by scanning electron microscopy (SEM)**

The morphology of microspheres (AMs or E-AMs) was examined by SEM at multiple magnifications. In addition, hydrogels were frozen and lyophilized, and the internal porous architecture was imaged by SEM to assess pore morphology and interconnectivity (Figure 1E–F).

**2.6 UV–Vis absorbance for emodin incorporation**

To corroborate emodin incorporation within the composite system, UV–Vis absorbance spectra of relevant formulations were recorded and compared (Figure 1G).

**2.7 Swelling and in vitro degradation**

Hydrogel discs (GM, AMs@GM, E-AMs@GM) were incubated in buffered solution at 37°C. For swelling, samples were weighed at predetermined time points after removing surface liquid, and the swelling ratio was calculated relative to the initial mass. For degradation, hydrogels were incubated for the indicated duration and mass loss was recorded over time to generate degradation profiles (Figure 1H–I).

**2.8 Qualitative adhesion assessment**

To assess interfacial adhesiveness, photocrosslinked hydrogels were applied onto diverse substrates (e.g., skin/tissue-mimicking surfaces and common engineering materials) and evaluated by qualitative handling/retention tests as illustrated in Figure 1J.

**2.9 Cell culture and material exposure**

Human bone marrow–derived mesenchymal stem cells (hBMSCs), RAW 264.7 macrophages, and C2C12 myoblasts were maintained under standard conditions (37°C, 5% CO₂) in complete growth medium. For biocompatibility and myogenic differentiation assays, hBMSCs and C2C12 myoblasts were seeded directly onto the surface of the crosslinked hydrogels at a density of 1 × 10⁴ cells/cm². For immunomodulation assays, RAW 264.7 macrophages were cultured with hydrogel-conditioned media. The conditioned media were prepared by incubating the sterilized hydrogels in complete medium (100 mg/mL) for 24 hours at 37°C, yielding an equivalent emodin concentration of 20 µM, which was used for all subsequent macrophage experiments.

**2.10 Live/Dead staining for cytocompatibility (hBMSCs)**

Cytocompatibility of GM, AMs@GM, and E-AMs@GM was assessed by Live/Dead staining, followed by fluorescence imaging and quantification of viable cells (Figure 2A–B).

**2.11 EdU incorporation assay for proliferation (hBMSCs)**

Cell proliferation was evaluated using an EdU incorporation assay. hBMSCs were cultured on the surface of the crosslinked hydrogels (Control, GM, AMs@GM, and E-AMs@GM) for 48 hours. Following this incubation, the cells were treated with 10 µM EdU solution for 2 hours at 37°C before fixation. Subsequently, EdU-positive nuclei were stained according to the manufacturer's protocol, imaged, and quantified to determine proliferative activity across groups (Figure 2C–D).

**2.12 Scratch wound-healing assay for migration (hBMSCs)**

For migration assessment, confluent hBMSC monolayers were scratched using a sterile tip. Images were recorded at baseline and subsequent time points, and wound closure was quantified to compare migratory capacity across groups (Figure 2E–G).

**2.13 Intracellular ROS detection under oxidative challenge (hBMSCs)**

To determine antioxidative capacity, hBMSCs were seeded at a density of 1 × 10⁴ cells/well and pre-incubated with the respective hydrogel treatments for 24 hours. Subsequently, the cells were subjected to an oxidative challenge using 200 µM H₂O₂ for 2 hours. Following the challenge, intracellular ROS levels were measured by incubating the cells with a fluorescent ROS probe (DCFH-DA, 10 µM for 30 minutes at 37°C). Fluorescence signals were then imaged and quantified (Figure 2H–I).

**2.14 Macrophage immunomodulation assays (RAW 264.7)**

**2.14.1 Immunofluorescence staining of polarization markers**

RAW 264.7 macrophages were incubated with the indicated hydrogel conditions, fixed, permeabilized, blocked, and stained for macrophage polarization markers. CD86 was used as an M1-associated marker and CD206 as an M2-associated marker. Representative images and quantification are shown in Figure 3A–D.

2.14.2 Cytokine quantification by ELISA

Cell supernatants were collected and analyzed by ELISA to quantify inflammatory cytokines (e.g., IL-6, TNF-α) and anti-inflammatory cytokine (IL-10), as presented in Figure 3E and 3I.

2.14.3 Gene expression analysis by RT–qPCR

Total RNA was extracted from macrophages, reverse transcribed, and subjected to RT–qPCR to quantify M1- and M2-associated transcripts (e.g., CD86, iNOS, TNF-α; CD206, Arg-1, IL-10). Data are shown in Figure 3F–H and 3J–L.

**2.15 Myogenic differentiation assays (C2C12)**

2.15.1 Myogenic induction

C2C12 myoblasts were cultured to near confluence and switched to differentiation medium to induce myotube formation. Cells were exposed to the indicated hydrogel conditions during differentiation.

2.15.2 Immunofluorescence staining and morphometric quantification

Differentiation was evaluated by immunofluorescence staining for Myosin Heavy Chain (MyHC/My32) and MyoD1 at day 7 and day 14. Myotube formation was quantified by myotube diameter and length (Figure 4).

**2.16 Murine VML model and hydrogel implantation**

A standardized murine volumetric muscle loss (VML) defect was created in the tibialis anterior (TA) muscle under anesthesia and sterile conditions. Briefly, a 1 cm skin incision was made to fully expose the TA muscle. A standardized muscle segment measuring 2×7×2 mm was excised to a depth of 2 mm using a scalpel, and the excised muscle was immediately weighed to confirm a removal weight of 15±1.5 mg per animal, ensuring consistent defect volume across all subjects. Animals were assigned to Sham, Model, and treatment groups receiving GM, AMs@GM, or E-AMs@GM. Hydrogels were placed into the defect site and photocrosslinked in situ using visible light illumination. The skin incision was subsequently closed with interrupted sutures. Tissues were harvested at 2 and 4 weeks post-implantation for histological.

**2.17 Histology (H&E and Masson’s trichrome)**

Harvested muscles were fixed, processed, sectioned, and subjected to hematoxylin and eosin (H&E) and Masson’s trichrome staining to assess tissue organization and collagen-rich fibrosis at 2 and 4 weeks (Figure 5B–C).

**2.18 Tissue immunofluorescence staining**

To evaluate regeneration, fibrosis, immune infiltration, and myogenic progenitors in vivo, sections were stained for:

- Laminin (myofiber boundary/architecture),
- Collagen I and α-SMA (fibrotic ECM and myofibroblast activation),
- CD86 and CD206 (macrophage phenotypes),
- PAX7 and MyoD1 (satellite cells and myogenic progenitors).
  Representative images correspond to Figure 5D–G, and quantitative summaries are shown in Figure 5H–K.

**2.19 Functional gait analysis by CatWalk XT**

Hindlimb locomotor function was assessed using the CatWalk XT automated gait analysis system. Mice were trained prior to data acquisition. Runs meeting predefined criteria (e.g., continuous traversal, stable speed) were analyzed. Representative footprint maps and dynamic pressure/force-related profiles are shown (Figure 6A–B), and multiple spatial/temporal parameters were extracted for statistical comparison (Figure 6C–J).

**2.20 In vivo biosafety evaluation**

Major organs (heart, liver, spleen, lung, kidney) were harvested and evaluated by H&E and Masson’s trichrome staining to assess systemic toxicity and off-target fibrosis. Representative results are shown in Supplementary Figure S1.

**2.21 Single-cell RNA sequencing (scRNA-seq) analysis**

Immediately after surgery, mouse samples were collected, and single-cell suspensions were prepared via mechanical dissociation and enzymatic digestion^1^. The concentration of the single-cell suspensions was adjusted as needed. Subsequently, the Chromium Next GEM single-cell 3’ GEM, library, and gel bead kit were used according to the manufacturer's instructions. All subsequent steps were performed according to the manufacturer's established protocols. The constructed libraries were sequenced using a NovaSeq sequencer (Illumina, USA). The Cell Ranger software (v7.1.0) was used to generate raw fastq files via its Cell Ranger mkfastq function; subsequently, the Cell Ranger count function was utilized to align and filter the raw fastq files with the reference genome, complete barcode counting and unique molecular identifier counting, and finally obtain the single-cell gene expression profiles^2–9^.

**2.22 Single-cell data processing**

The original gene expression data were imported into R software (v4.4.1) and analyzed using the Seurat R package (v4.3.0). After removing doublets, quality control was performed using the DoubletFinder R package (v2.0.3), with the following screening criteria: (1) 300 < nFeature < 7500; (2) 500 < nCount < 100,000; (3) mitochondrial gene expression does not exceed 25% of the total gene expression in a single cell; (4) erythroid gene expression does not exceed 5% of the total number of genes in the cell. For the remaining cells, the gene expression matrix of the remaining cells was generated using the "NormalizeData" and "ScaleData" functions in Seurat (v4.3.0) through logarithmic normalization and linear regression techniques. Subsequently, the "Find Variable Features" function was used to screen the top 2000 genes with the highest variability. After principal component analysis and dimensionality reduction, the Harmony package (v0.1.1) was used for batch effect correction, and the first 30 principal components were selected for subsequent analysis^10,11^.

**2.23 Cell type identification**

The first 30 principal components were used as input, and clustering was performed using the "FindNeighbors" and "FindClusters" functions to identify different cell clusters. Based on published literature and known cell marker genes from the CellMarker database, marker genes associated with different cell types were identified These marker genes were used to annotate cell clusters, identify different cell types, and analyze their distribution patterns and proportions. In addition, to explore macrophage heterogeneity, macrophages were subjected to secondary clustering, and the resulting subpopulations were annotated based on their specific gene expression profiles.

**2.24Functional enrichment analysis**

The Wilcoxon rank-sum test was performed using Seurat's "FindAllMarkers" function to evaluate differentially expressed genes (DEGs) in different cell clusters^12^. The ClusterProfiler R package (v4.6.2) and SCP package(v0.4.8) were used to perform Gene Ontology (GO)^13^, Kyoto Encyclopedia of Genes and Genomes (KEGG)^14,15^, and Gene Set Enrichment Analysis (GSEA) enrichment analyses on DEGs from different cell types^16–18^. The significance of GO terms was determined based on a corrected p-value < 0.05.

**2.25 AUCell rating**

The scgmt R package (v0.0.3, https://github.com/ZhaoLabs-SJTU/scgmt) was used to calculate the activity score of the characteristic gene set, and AUCell (v1.20.2) was selected as the scoring method. AUCell is a method for identifying cells with active gene sets in single-cell RNA sequencing data. It takes a predefined gene set as input and outputs the activity score of the corresponding gene set for each cell. Further quantitative analysis of pathway activity was performed using AUCell^19,20^.

**2.26 Cell development trajectory inference**

Stem cell characteristics were assessed using the CytoTRACE package (version 0.3.3), which reveals the temporal progression of cell differentiation^21^. Pseudo-temporal trajectory analysis was performed using the Monocle R package (v2.24.0), with dimensionality reduction using uniform manifold approximation and projection (UMAP) methods, and visualization via the "PLOT-CELL-TRACTURE" function^22^. Next, different cell subpopulations were sorted according to the pseudo-temporal order, and genes exhibiting co-expression changes along the pseudo-temporal trajectory were screened and displayed using a pseudo-temporal heatmap.

Following this, the Slingshot R package (v2.6.0) was used to construct a minimum spanning tree using the "getLineage" function to infer the cell developmental lineage structure, and the branch of each lineage was modeled by fitting the simultaneous principal curve^23^; further, the "getCurves" function was used to calculate the gene expression changes of different lineages along the pseudo-temporal trajectory to obtain smooth trajectory curves.

**2.27 Cell communication**

The CellChat R package (v1.6.1) was used to infer complex intercellular interactions associated with skeletal muscle injury at the ligand-receptor level, and corresponding regulatory networks were constructed^24,25^. Potential interactions between different cell types were predicted, and the "netVisualDiffInteraction" function was used to visualize differences in intercellular communication strength. The number of communication patterns was assessed using the "IdentifyCommunicationPatterns" function, and the analysis results were further presented in the form of hierarchical plots and ligand-receptor heatmaps using the "netVisual_circle" function. A *p*-value < 0.05 was used as a significance threshold to screen for significant cell-cell communication relationships between different cell types.

Furthermore, ring plots, bubble plots, and violin plots were used to visualize the input and output signals of all cell types. The analysis focused on the communication between macrophage and other cell types and their related signaling pathways to explore the synergistic regulatory mechanisms of signaling pathways among different cell populations^26–29^.

**2.28 SCENIC analysis**

To investigate the top five transcription factors (TFs) with the most significant expression changes in macrophage subtypes^30^, single-cell regulatory network inference and cluster analysis were performed using the SCENIC package (v1.3.1) in a Python (v3.7) environment^31^. First, GRNBoost was used to identify potential target genes for each transcription factor; next, DNA motif analysis was used to determine possible direct binding targets, and co-expression modules were constructed, while the regulatory weights between transcription factors and their target genes were evaluated. Then, AUCell was used to score the activity of regulators in each cell, and the top five transcription factors with the highest activity scores were selected^32^.

**2.29 Quantification and statistics**

This study used R and Python software for database data analysis. Image-based quantification (viability, EdU positivity, scratch closure, fluorescence intensity/positive area, histological fibrosis area, and morphometrics of myotubes) was performed using standard image analysis software (ImageJ/Fiji) with consistent thresholds across groups. Data are presented as mean ± standard deviation (SD). All sample sizes (n) reported in the figure legends represent independent biological replicates. Statistical analyses were performed using GraphPad Prism software. Comparisons between two independent groups were evaluated using an unpaired, two-tailed Student's t-test. Comparisons among three or more groups were analyzed using One-way or Two-way Analysis of Variance (ANOVA) followed by Tukey's post hoc test for multiple comparisons. A two-tailed P < 0.05 was considered statistically significant (*P < 0.05, **P < 0.01, ***P < 0.001, ****P < 0.0001).

**Reference**

1.	Lei MK, Ong ML, Simons RL, Beach SRH. Biological aging and its association with serum neurofilament light chain levels in middle-aged african americans: a prospective observational study. *Aging Adv*. 2025;2(1):1-8. doi:10.4103/AGINGADV.AGINGADV-D-24-00021

2.	Wang Y, Zheng M, Zhang Y, et al. Single-cell transcriptomic analysis of the immune response to COVID-19 and tuberculosis coinfection. *Exploration*. 2025;5(5):20240022. doi:10.1002/EXP.20240022

3.	Xia Y, Wang Y, Shan M, Hao Y, Liang Z. Decoding the molecular landscape of keloids: new insights from single-cell transcriptomics. *Burns Trauma*. 2023;11:tkad017. doi:10.1093/burnst/tkad017

4.	Du H, Li S, Lu J, et al. Single-cell RNA-seq and bulk-seq identify RAB17 as a potential regulator of angiogenesis by human dermal microvascular endothelial cells in diabetic foot ulcers. *Burns Trauma*. 2023;11:tkad020. doi:10.1093/burnst/tkad020

5.	Cheng XC, Tong WZ, Rui W, Feng Z, Shuai H, Zhe W. Single-cell sequencing technology in skin wound healing. *Burns Trauma*. 2024;12:tkae043. doi:10.1093/burnst/tkae043

6.	Yang L, He YT, Dong S, et al. Single-cell transcriptome analysis revealed a suppressive tumor immune microenvironment in EGFR mutant lung adenocarcinoma. *J ImmunoTher Cancer*. 2022;10(2):e003534. doi:10.1136/jitc-2021-003534

7.	Shi J, Liu J, Tu X, et al. Single-cell immune signature for detecting early-stage HCC and early assessing anti-PD-1 immunotherapy efficacy. *J ImmunoTher Cancer*. 2022;10(1):e003133. doi:10.1136/jitc-2021-003133

8.	Zhao Z, Cai H, Nie W, et al. Ectopic expression of GDF15 in cancer-associated fibroblasts enhances melanoma immunosuppression via the GFRAL/RET cascade. *J ImmunoTher Cancer*. 2025;13(6):e011036. doi:10.1136/jitc-2024-011036

9.	Franken A, Van Mol P, Vanmassenhove S, et al. Single-cell transcriptomics identifies pathogenic T-helper 17.1 cells and pro-inflammatory monocytes in immune checkpoint inhibitor-related pneumonitis. *J ImmunoTher Cancer*. 2022;10(9):e005323. doi:10.1136/jitc-2022-005323

10.	Liu Y, Chen M, Zhang L, et al. Comparisons in microglial responses to ischemic–hypoxic brain injury in neonatal and adult mice analyzed by transcriptome integration and deconvolution technologies. *Adv Technol Neurosci*. 2025;2(4):161-172. doi:10.4103/ATN.ATN-D-25-00004

11.	Zhang H, Zhang G, Xu P, et al. Optimized dynamic network biomarker deciphers a high-resolution heterogeneity within thyroid cancer molecular subtypes. *Med Res*. 2025;1(1):10-31. doi:10.1002/mdr2.70004

12.	Zha X. Bioinformatics analysis techniques identify the ferroptosis-related gene MYC as a potential therapeutic target for spinal cord injury: an observational study based on the GEO database. *Adv Technol Neurosci*. 2025;2(2):59-71. doi:10.4103/ATN.ATN-D-24-00026

13.	Jin X, Ji X, Yin H, et al. Identification of potential targets of stress cardiomyopathy by a machine learning algorithm. *Cardiovasc Innov Appl*. 2024;9(1). doi:10.15212/CVIA.2024.0011

14.	Li S, Yao J, Zhang S, et al. Prognostic value of tumor-microenvironment-associated genes in ovarian cancer. *BIO Integr*. 2023;4(3). doi:10.15212/bioi-2022-0008

15.	Li Y, Du J, Liu B, She Q. Identifying key genes and related molecules as potential biomarkers in human dilated cardiomyopathy by comprehensive bioinformatics analysis. *Cardiovasc Innov Appl*. 2023;8(1). doi:10.15212/CVIA.2023.0018

16.	Wang X, Li J, Ning J. Comprehensive multi-omics integration reveals B cell-derived ELL2 as a novel diagnostic and prognostic biomarker in sepsis. *Med Res*. 2025;1(2):170-174. doi:10.1002/mdr2.70010

17.	Zhang J, Li H, Tao W, Zhou J. GseaVis: an R package for enhanced visualization of gene set enrichment analysis in biomedicine. *Med Res*. 2025;1(1):131-135. doi:10.1002/mdr2.70000

18.	Chen Z, Gao Q, Qiu J, et al. Genetic analysis reveals key regulatory axis in aortic dissection: CBL regulated by HOXB13 and microRNA-1321. *Cardiovasc Innov Appl*. 2024;9(1). doi:10.15212/CVIA.2024.0034

19.	Zhao Z, Cai H, Zhao Z, et al. Cancer-associated fibroblast-derived GDF15 induces oxidative stress and neutrophil infiltration in head and neck squamous cell carcinoma through the PI3K/AKT/STAT3 axis cascade. *Research*. 2025;8:0901. doi:10.34133/research.0901

20.	Wu J, Liu W, Qiu X, et al. A noninvasive approach to evaluate tumor immune microenvironment and predict outcomes in hepatocellular carcinoma. *Phenomics*. 2023;3(6):549-564. doi:10.1007/s43657-023-00136-8

21.	Lou W, Qiu X, Qin Y, Lu Y, Cao Y, Lu H. 3D-printed advanced scaffold armed with exosomes derived from human skeletal stem cell identified by single-cell RNA sequencing enhances osteochondral regeneration. *Bioact Mater*. 2025;51:231-256. doi:10.1016/j.bioactmat.2025.04.028

22.	Liu Y, Niu H, Ren J, et al. Single-cell RNA sequencing reveals abnormal transcriptome signature of erythroid progenitors in pure red cell aplasia. *Genes Dis*. 2024;11(1):49-52. doi:10.1016/j.gendis.2023.03.002

23.	Yang X, Huang M, Chen H, et al. Biomaterial-mediated cell atlas: an insight from single-cell and spatial transcriptomics. *Bioact Mater*. 2025;54:1-33. doi:10.1016/j.bioactmat.2025.07.047

24.	Wu J, Hu N, Guo B, et al. Single-cell transcriptomics systematically discloses the immune-inflammatory responses of peripheral blood mononuclear cells for diabetic nephropathy. *Genes Dis*. 2024;11(4):101033. doi:10.1016/j.gendis.2023.06.007

25.	Xie J, Yang A, Liu Q, et al. Single-cell RNA sequencing elucidated the landscape of breast cancer brain metastases and identified ILF2 as a potential therapeutic target. *Cell Proliferation*. 2024;57(11):e13697. doi:10.1111/cpr.13697

26.	Li J, Zhao C, Xu Y, et al. Remodeling of the osteoimmune microenvironment after biomaterials implantation in murine tibia: single-cell transcriptome analysis. *Bioact Mater*. 2023;22:404-422. doi:10.1016/j.bioactmat.2022.10.009

27.	Mishra S, Deep G. Mitochondria-derived vesicles: potential nano-batteries to recharge the cellular powerhouse. *Extracell Vesicles Circ Nucleic Acids*. 2024;5(2):271-275. doi:10.20517/evcna.2023.71

28.	Colvett I, Saternos H, Coughlan C, Vielle A, Ledreux A. Extracellular vesicles from the CNS play pivotal roles in neuroprotection and neurodegeneration: lessons from in vitro experiments. *Extracell Vesicles Circ Nucleic Acids*. 2023;4(1):89-106. doi:10.20517/evcna.2023.07

29.	Chakraborty R, Belian S, Zurzolo C. Hijacking intercellular trafficking for the spread of protein aggregates in neurodegenerative diseases: a focus on tunneling nanotubes (TNTs). *Extracell Vesicles Circ Nucleic Acids*. 2023;4(1):27-43. doi:10.20517/evcna.2023.05

30.	Yu A, Fu J, Yin Z, et al. Continuous expression of interferon regulatory factor 4 sustains CD8+ T cell immunity against tumor. *Research*. 2023;6:0271. doi:10.34133/research.0271

31.	Huang L, Ye L, Li R, et al. Dynamic human retinal pigment epithelium (RPE) and choroid architecture based on single-cell transcriptomic landscape analysis. *Genes Dis*. 2023;10(6):2540-2556. doi:10.1016/j.gendis.2022.11.007

32.	Chen Y, Sun Y, Luo Z, et al. Potential mechanism underlying exercise upregulated circulating blood exosome miR-215-5p to prevent necroptosis of neuronal cells and a model for early diagnosis of alzheimer’s disease. *Front Aging Neurosci*. 2022;14:860364. doi:10.3389/fnagi.2022.860364

**S**

**Supplementary Figure 1. In vivo biosafety assessment of the hydrogel formulations by histopathology.**

Representative H&E and Masson’s trichrome staining of major organs harvested from mice treated with GM, AMs@GM, or E-AMs@GM at the indicated time point after implantation. Sections from the heart, liver, spleen, lung, and kidney were examined to evaluate potential systemic toxicity, inflammatory lesions, and abnormal collagen deposition/fibrosis. No overt pathological abnormalities were observed across groups. Scale bars are indicated in the images.

**Supplementary Figure 2. Visualization of single-cell microenvironment and macrophages in mouse skeletal muscle tissue after E-AMs@GM intervention.**

**(A)** The heatmap showed the expression of the Top5 DEGs in cell types.

**(B)** UMAP clustering plots of mouse skeletal muscle cells. From top to bottom, the pie plot sequentially showed the grouping origins (model group and E-AMs@GM group) and cell cycle stages (G1, G2M, and S) for each cell type.

**(C)** The Ro/e preference analysis heatmap showed the preference distribution of the 13 cell types in the model group and the E-AMs@GM group.

**(D)** The Ro/e preference analysis heatmap showed the preference distribution of the 13 cell types across different cell cycle stages.

**(E)** Bar plots illustrated the distribution of various cell types across the cell cycle phases (G1, G2M, and S).

**(F)** The volcano plot showed the Top5 DEGs in macrophages; DEGs above the horizontal line were upregulated, whereas those below the horizontal line were downregulated.

**(G)** Enrichment network plot illustrated the functional clusters and interrelationships of enriched pathways in macrophages.

**(H)** GO functional enrichment analysis of macrophages. The bar plot displayed significantly enriched terms categorized into Biological Process, Cellular Component, and Molecular Function. The number of genes in the "Detected" and "Enriched" categories for each GO term was indicated.

**(I)** UMAP clustering of mouse skeletal muscle macrophage subtypes combined with pie plots. From left to right, the relative proportions of experimental groups (model and E-AMs@GM) and cell cycle phases (G1, G2M, and S) across different cell types were displayed.

**(J)** The proportion plot showed the proportion of the four macrophage subtypes across the cell cycle.

**(K)** The Ro/e preference analysis heatmap showed the preferred distribution of the four macrophage subtypes across the cell cycle.

**(L)** The bar plots showed the expression levels of the signature marker genes (*Ccr2*, *Mmp12*, *Gdf15*, and *Arg1*) across the four macrophage subtypes.

**Supplementary Figure 3. Macrophages Subtype-Specific Differential Expression Characteristics and Functional Pathway Enrichment Analysis**

**(A)** The volcano plots showed the top five differentially expressed genes that were upregulated and downregulated in C0 *Ccr2⁺* macrophages, C2 *Gdf15⁺* macrophages, and C3 *Arg1⁺* macrophages.

**(B)** The word clouds showed the basic functional enrichment of C0 *Ccr2⁺* macrophages, C2 *Gdf15⁺* macrophages, and C3 *Arg1⁺* macrophages.

**(C)** The bar charts showed the GO-BP functional enrichment analysis results of C0 *Ccr2⁺* macrophages, C2 *Gdf15⁺* macrophages, and C3 *Arg1⁺* macrophages.

**(D)** GSEA plots for C0 *Ccr2⁺* macrophages, C2 *Gdf15⁺* macrophages, and C3 *Arg1⁺* macrophages were shown. C0 showed a positive enrichment trend in the "Aminoglycan biosynthetic process" and "miRNA metabolic process"; C2 showed a positive enrichment trend " in thePost-Golgi vesicle-mediated transport" and "Immunoglobulin-mediated immune response"; C3 showed a positive enrichment trend in the "Positive regulation of telomerase RNA localization to Cajal body" and "Regulation of establishment of protein localization to chromosome".

**(E)** GO enrichment analysis showed the biological enrichment pathways for each macrophage subtype.

**(F)** The bubble plots showed the expression of genes related to M1 and M2 functions in macrophage subtypes (C0–C3).

**(G)** The bubble plots showed the expression of genes related to efferocytosis and anti-inflammatory functions in macrophage subtypes (C0–C3).

**Supplementary Figure 4. Macrophages Subtype-Specific Regulatory Modules, Key Transcription Factors, and Receptor-Ligand Interaction Network Analysis**

**(A)** The hierarchical clustering heatmap showed the similarity among macrophage regulatory modules (M1-M4) based on regulatory activity scores. Colors from light to dark indicated regulatory activity from low to high.

**(B–C)** Scatter plots showed the regulatory activity scores across different macrophage subtypes within the four regulatory modules (M1–M4), and

specifically showed the differential regulon activity scores of the M4 regulatory module between the model group and the E-AMs@GM group.

**(D)** The scatter plot showed the distribution of specificity scores for TFs within the M4 regulatory module, highlighting key transcription factors highly correlated with the M4 module; other factors were categorized as Others.

**(E)** The heatmap showed the scores of the Top5 TFs across the four macrophage subtypes.

**(F)** The dot plot showed the top five transcription factors in the C1 *Mmp12⁺* macrophage specificity score.

**(G–I)** The distribution of *Ets2*, *Fosl2*, *Hif1a*, *Cebpb*, and *Churc1* across different macrophage subtypes and cell sample sources was calculated using the AUCell algorithm.

**(J)** The circle plots showed the interaction network of macrophages subtypes with other cell types across four ligand-receptor pairs

**(K–L)** UMAP and bar plots showed the expression of receptors (*Cd44*, *Itgb1*, *Itgav*, *Itga5*, and *Itgb5*) in different macrophage subtypes.

**Supplementary Table 1: Antibody catalog number.**

| Antibody | Source | Catalog No. | Type of Ab | Dilution | M.W.(kD) |
| --- | --- | --- | --- | --- | --- |
| iNOS | Abcam | ab178945 | Rabbit mAb | 1:200(IF) | 131 |
| CD86 | Abcam | ab220188 | Mouse mAb | 1:100(IF) | 38 |
| CD163 | Abcam | ab156769 | Mouse mAb | 1:100(IF) | 125 |
| F4/80 | Abcam | ab6640 | Rat mAb | 1:200(IF) | 97 |
| Arg1 | Affinity | DF6657 | Rabbit PAb | 1:500(IF) | 35 |
| MY-32 | Abcam | ab51263 | Mouse mAb | 1:500(IF) | 227 |
| MYOD1 | Proteintch | 18943-1-AP | Rabbit PAb | 1:200(IF)  1:1000(WB) | 35 |
| Pax7 | Santa Cruz | SC-81648 | Mouse mAb | 1:20(IF)  1:500(WB) | 55 |
| Collagen 1 | Affinity | AF7001 | Rabbit PAb | 1:200(IF) | 129 |
| a-SMA | Abcam | ab7817 | Mouse mAb | 1:200(IF) | 42 |
| Lamin B1 | Abcam | ab16048 | Rabbit PAb | 1:100(IF) | 66 |
| Myf5 | Abcam | ab125078 | Rabbit mAb | 1:3000(WB) | 37 |
| Notch1 | Abcam | ab52627 | Rabbit mAb | 1:1000(WB) | 125 |
| Acox1 | Affinity | DF12046 | Rabbit PAb | 1:1000(WB) | 50 |
| CPT1A | Affinity | DF12004 | Rabbit PAb | 1:1000(WB) | 88 |
| GAPDH | Proteintch | 60004-1-Ig | Mouse mAb | 1:10000(WB) | 36 |
| Tubulin | Proteintch | 66240-1-Ig | Mouse mAb | 1:10000(WB) | 55 |
| CD86 FITC | eBioscience | 11-0862-82 | Rat mAb | 0.125 µg/test | (Flow Cyt) |
| CD206 APC | eBioscience | 17-2061-80 | Rat mAb | 0.25 µg/test | (Flow Cyt) |
| F4/80 PE-Cyanine5 | eBioscience | 15-4801-80 | Rat mAb | 0.25 µg/test | (Flow Cyt) |
| Goat Anti-Rabbit IgG H&L (Alexa Fluor® 488) | Abcam | ab150077 | Secondary antibody | 1:500(IF) | - |
| Goat Anti-Mouse IgG H&L (Alexa Fluor® 488) | Abcam | ab150113 | Secondary antibody | 1:500(IF) | - |
| Goat Anti-Rabbit IgG H&L (Alexa Fluor® 594) | Abcam | ab150080 | Secondary antibody | 1:500(IF) | - |
| Goat Anti-Mouse IgG H&L (Alexa Fluor® 594) | Abcam | ab150116 | Secondary antibody | 1:500(IF) | - |
| Goat Anti-Rat IgG H&L (Alexa Fluor® 594) | Abcam | ab150160 | Secondary antibody | 1:500(IF) | - |
| Cy5 conjugated Goat Anti-mouse IgG (H+L) | Servicebio | GB27301 | Secondary antibody | 1:300(IF) | - |
| HRP-conjugated Goat Anti-Mouse IgG(H+L) | Proteintch | SA00001-1 | Secondary antibody | 1:3000(WB) | - |
| HRP-conjugated Goat Anti-Rabbit IgG(H+L) | Proteintch | SA00001-2 | Secondary antibody | 1:3000(WB) | - |
